# Promoting quality use of medicines in South-East Asia: reports from country situational analyses

**DOI:** 10.1186/s12913-018-3333-1

**Published:** 2018-07-05

**Authors:** Kathleen Anne Holloway, Anita Kotwani, Gitanjali Batmanabane, Budiono Santoso, Sauwakon Ratanawijitrasin, David Henry

**Affiliations:** 1 International Institute of Health Management Research, Jaipur, India; 2 Institute of Development Studies, University of Sussex, Brighton, BN1 9RE UK; 3 Department of Pharmacology, Vallabhbhai Patel Chest Institute, University of Delhi, New Delhi, India; 4 All India Institute of Medical Sciences, Bhubaneswar, India; 5 Independent Consultant in Medicines Policy, Yogyakarta, Indonesia; 6 Faculty of Social Sciences and Humanities, Mahidol University, Salaya, Nakornpathom Thailand; 7 Bond University, Gold Coast, QLD Australia; 8 University of Toronto, Toronto, Canada

**Keywords:** South-East Asia, Quality use of medicines, Essential medicines policy

## Abstract

**Background:**

Irrational use of medicines is widespread in the South-East Asia Region (SEAR), where policy implementation to encourage quality use of medicines (QUM) is often low. The aim was to determine whether public-sector QUM is better in SEAR countries implementing essential medicines (EM) policies than in those not implementing them.

**Methods:**

Data on six QUM indicators and 25 EM policies were extracted from situational analysis reports of 20 country (2-week) visits made during 2010–2015. The average difference (as percent) for the QUM indicators between countries implementing versus not implementing specific policies was calculated. Policies associated with better (> 1%) QUM were included in regression of a composite QUM score versus total number of policies implemented.

**Results:**

Twenty-two policies were associated with better (> 1%) QUM. Twelve policies were associated with 3.6–9.5% significantly better use (*p* < 0.05), namely: standard treatment guidelines; formulary; a government unit to promote QUM; continuing health worker education on prescribing by government; limiting over-the-counter (OTC) availability of systemic antibiotics; disallowing public-sector prescriber revenue from medicines sales; not charging fees at the point of care; monitoring advertisements of OTC medicines; public education on QUM; and a good drug supply system. There was significant correlation between the number of policies implemented out of 22 and the composite QUM score (*r* = 0.71, r^2^ = 0.50, *p* < 0.05).

**Conclusions:**

Country situational analyses allowed rapid data collection that showed EM policies are associated with better QUM. SEAR countries should implement all such policies.

**Electronic supplementary material:**

The online version of this article (10.1186/s12913-018-3333-1) contains supplementary material, which is available to authorized users.

## Background

Inappropriate (irrational, incorrect, improper, poor quality) use of medicines is a serious public health problem world-wide [1–5] that wastes resources and may result in treatment failure and avoidable adverse drug events, including antimicrobial resistance [6–8], hospitalisation and death [9–11]. The World Health Organisation (WHO) has been promoting the concept of essential medicines and a range of policies to promote better quality (rational) use of medicines (QUM) for many years [12, 13]. The extent to which countries, including many in South-East Asia, monitor use or implement these recommended policies vary greatly [14, 15]. A review of interventions to promote better QUM in low and middle-income countries found that relatively few had been implemented and that most were small scale of short duration with small or modest effect [4, 5].

Analysis of secondary data on public-sector medicines use in primary care (from WHO’s database of medicines use surveys) and policy implementation as reported by Ministries of Health (MOH) (from questionnaires sent to Ministries of Health by WHO) showed that many essential medicines policies are associated with better QUM and that the more policies are implemented the better the use [16, 17]. Policies most strongly associated with QUM were: undergraduate training of doctors and nurses in standard treatment guidelines, the ministry of health having a unit promoting QUM, and provision of essential medicines free at point of care to all patients [16, 17].

The need for an integrated health systems approach, incorporating regular monitoring of medicines use and the sustainable implementation of multiple policies has long been recognised [18, 19]. However, development of such an approach remains elusive in many low and middle-income countries, where data are scant, infrastructure is lacking and responsibility for medicines management often falls between different departments with no clear accountability [20].

Since 2010 South-East Asian countries of the WHO have been undertaking 4-yearly rapid situational analyses of how medicines are used and managed with a view to developing a more integrated, coordinated health systems approach to promoting better QUM [21]. This process consists of rapid systematic data collection on medicines use and policy implementation by a multidisciplinary government team of four to eight people over 2 weeks using a predesigned workbook tool and ending with a national workshop to identify priorities for action [21]. During 2010–2015, all 11 jurisdictions (all South-East Asia countries including two Indian States) had conducted at least one situational analysis; eight had conducted two situational analyses separated by a period of 4 years; and the reports published on the WHO’s South-East Asia Regional Office (SEARO) website after government approval [21].

The aim of this paper was to investigate associations between the adoption (implementation or partial implementation) of national policies intended to improve QUM and patterns of medicine use in WHO South-East Asian countries. The analysis relies on data on medicines use in public-sector primary care, and policy implementation, extracted from the country situational analysis reports [21]. Specific objectives were to establish for the WHO South-East Asia region:

1. which policies are associated with better QUM,
2. whether the implementation of more policies is associated with better QUM, and
3. whether there was any improvement in policy implementation and QUM in countries that had undertaken two situational analyses.

## Methods

The country reports of the situational analyses published on the website of the WHO Regional Office for South-East Asia (WHO/SEARO) [21] were reviewed and data extracted on QUM in public-sector primary care and the implementation of policies to encourage appropriate use. The methods for conducting country situational analyses have been described elsewhere [21, 22] and are summarised in Table 1. Briefly, data on policy implementation was collected by interviews of health staff and observation, and data on QUM by prescription survey following the International Network for the Rational Use of Drugs (INRUD)/WHO methodology [23, 24] at all the health facilities visited. The methods used to analyse the extracted data followed similar methods used by first author in analysing global data and are described elsewhere [16, 17] and summarised below.

**Table 1:** Summary of methods used in a country situational analysis

Background	
Development of the situational analysis approach in South-East Asia was requested by Member States [38, 39] and involves the systematic collection of data by a government multi-disciplinary team over 2 weeks using a pre-designed workbook tool [21] and supervised by WHO. The workbook tool (Additional file 2) builds on other tools [14, 23, 24] and was developed by WHO/SEARO during situational analyses done in 11 countries during 2010–13 and piloted for use by government staff in eight countries during 2014–15.	
Methods	
Visits are made to: • all major MOH units and other agencies responsible for medicines management (supply, selection, use, regulation, policy, insurance and professional training) to understand what policies are in place and what each unit does. • healthcare facilities, aiming to visit 20 facilities, two of each type of public facility (primary care centres and sub-centres, secondary and tertiary hospitals, with half of the facilities being primary care centres) plus private pharmacies (results not reported in this paper) in at least two provinces/regions, as selected by the MOH.	
Data collection and Analysis	
At the central level, staff are interviewed about the health system, what their unit does and what policies are in place. At each health facility (whether hospital or health centre), the team reviews 30 primary care outpatient encounters (using whatever documentation is available at the facility, e.g. prescriptions held in the pharmacy or by the patient, paper slips in the pharmacy, patient records, or outpatient registers). The means for standard indicators of medicines use [23, 24] are calculated for each facility and each category of facility. Also, antibiotic use in 30 outpatient cases of upper respiratory tract infection is reviewed, although this is difficult in some countries where diagnosis is not recorded on the prescription. The basis for a diagnosis of upper respiratory tract infection is also recorded e.g. acute viral respiratory infection, pharyngitis, sore throat, rhinitis, runny nose, cough, cold, otitis media, earache, sinusitis, acute laryngitis and acute bronchitis. The medicines’ supply and regulatory systems are also reviewed and health workers interviewed about medicine management policy implementation. A descriptive analysis is done each day and presented by the team at a national workshop at the end of 2 weeks, and a country report published on the WHO/SEARO website after government approval [21, 38].	

A dataset was created (Additional file 1) consisting of six standard QUM indicators [16, 17] and indicators for implementation of 25 policies hypothesised to influence medicines use [14–17], derived from data collected during 20 country situational analysis visits. Since different provinces or regions and different healthcare facilities were visited in the countries where two situational analyses were done, and the visits were separated by 3–5 years (during which time the implementation of some policies changed), these situational analyses were treated as separate records (country-year jurisdictions) in the dataset.

The QUM indicators (described in Table 2, together with the direction of more appropriate use) are all expressed as proportions and include all the indicators measured in the country situational analyses apart from one (the average number of drugs per patient). In the situational analysis reports [21], QUM indicators were reported as an average for each facility type, but for this analysis one result per QUM indicator was calculated, this being the average across all facilities.

**Table 2:** Quality Use of Medicines (QUM) indicators and direction of better use

QUM Indicator	Direction of better use
% Upper Respiratory Tract Infection (URTI) cases (patients) treated with antibiotics	Less
% cases (patients) treated with antibiotics	Less
% prescribed medicines from the national Essential Medicines List (EML)	More
% medicines prescribed by generic name	More
% cases (patients) treated with multivitamins	Less
% cases (patients) treated with an injection	Less

Six standard indicators of quality of medicines use [23, 24] expressed as proportions and reported in 85–100% of the situational analyses

The policy indicators (described in Table 3) are all expressed as categorical yes/no variables and include all those policies hypothesised to improve the appropriate use of medicines [16, 17] for which data were available in the country situational reports [21] and for which there were countries with and without the policy (for comparison). Some policies hypothesised to influence prescribing were not implemented by any country (e.g. monitoring all drug promotional activities), but if possible, similar more limited policies were substituted (e.g. monitoring of advertisements restricted to over-the-counter drugs). Where policy implementation was expressed as a range of values, the policy was converted to a yes/no value. For example, “some” public education and “some” health worker training was regarded as “yes” when assessing whether a country had implemented these policies. Details of decisions on whether a policy was marked as present or not can be seen in Table 3. Since country wealth may be a potential confounder (being associated with both better QUM and greater policy implementation), data on gross national income per capita (GNIpc) were extracted for each country in the year of the situational analysis from the United Nations (UN) Country Profile Data [25].

**Table 3:** Medicine Policy variables with information on how a policy was judged to be present or not

Policies recommended to improve medicines use^a^		Criteria to determine whether a policy was adopted (implemented or partially implemented) in a country
National structures, medicines policies and monitoring		
1	National MOH unit on promoting rational use of medicines	Policy was marked “yes” if there was any unit, even if very small and consisting of only 1–2 persons, or an executive committee with responsibility for promoting quality use of medicines.
2	Presence of a Drug and Therapeutic Committee (DTC) in most referral hospitals	Policy was marked “yes” if more than half of referral hospitals visited had a DTC which had met in the last year (even if not very active) and there was an MOH mandate for DTCs.
3	National strategy to contain antimicrobial resistance	Policy was marked “yes” if there was any policy document endorsed by MOH on AMR containment.
4	Presence of National Drug Information Centre	Policy was marked “yes” if any national drug information centre existed, even if the centre was not very active and did not offer 24-hour emergency information.
5	Prescription audit in the last 2 years	Policy was marked “yes” even if the audit had only been undertaken in the health facilities of some districts, but including at least one of the districts visited during the situational analysis.
Educational policies		
6	Undergraduate training of prescribers on the National Essential Medicines List (EML)	Policy was marked “yes” even if only some training institutions included the EML in the curriculum.
7	Undergraduate training of prescribers on the National Standard Treatment Guidelines (STGs)	Policy was marked “yes” even if only some training institutions included the STG in the curriculum.
8	Continuing medical education (CME) of prescribers by MOH	Policy was marked as “yes” even if only some prescribers received CME on general prescribing in adults and/or children. The Antibiotic SMART Use program in Thailand, INRUD training activities in Nepal and the training activities of the National Institute of Health (INS) in Timor-Leste are examples of CME by the MOH [21, 37].
9	Public education on medicines use in last 2 years	Policy was marked “yes” if any district populations had received public education.
Managerial Policies		
10	National Essential Medicines List updated in the last 2 years	Was not hypothesized to influence antibiotic use.
11	National Standard Treatment Guidelines updated in the last 2 years	Policy was marked” yes” if there was any kind of officially published book containing national treatment guidelines, but not for disease protocols on posters or pamphlets.
12	National Standard Treatment Guidelines (STGs) found in some health facilities (indicator of STG implementation).	Policy was marked “yes” if the national STGs (published book) were observed in more than two facilities visited.
13	National Formulary available	Policy was marked “yes” if any national formulary was observed in any facility.
14	Generic prescribing policy in public sector	Policy was marked “yes” if there was any initiative described to encourage generic prescribing. Was not hypothesized to influence antibiotic use.
15	Generic substitution in public sector	Policy was marked “yes” if generic substitution was both legal and seen to occur. Was not hypothesized to influence antibiotic use.
16	Prescriber workload low or moderate	Low/moderate workload defined as less than 60 patients per prescriber per day, as reported by prescribers or as observed in patient registers.
Supply system		
17	Public sector procurement limited to only EML medicines	Policy was marked “yes” if public sector procurement limited to EML medicines was reported at the central level and observed at the health facilities visited. Indicator of implementation of the EML.
18	No medicines stock-out problems reported in the health facilities visited	Policy was marked “yes” if health workers at the facilities visited stated that there were no stock-out problems. Indicator of the quality of the supply system which may impact on use.
Economic Policies		
19	NO Drug sales revenue used to supplement prescriber income	Policy was marked “no” if prescribers were observed selling drugs in the public sector, as was the case in one country in 1 year.
20	No registration or consultation fee	All countries stated that they dispensed drugs free of charge to all patients in public facilities if medicines were available, but some charged registration or consultation fees which could be perceived by patients as payment for treatment.
21	No user fee or copayment at the point of care	Although all countries officially dispensed drugs free of charge in public facilities, some types of facility, generally hospitals, charged a user fee or co-payment for drugs at the point of care.
Regulatory policies		
22	Systemic antibiotics generally not available over-the-counter (OTC)	Systemic antibiotics could be got OTC in all countries but were generally unavailable in Bhutan and DPR Korea where the private sector is very small, and effort is made to enforce the drug schedules.
23	Regulation of advertisements for OTC drugs medicines	No countries were monitoring all drug promotional activities, but some did monitor advertising of OTC drugs.
Human resource policies		
24	Prescribing by doctors (as opposed to other staff) in public primary care	Policy was marked “yes” if doctors were observed to be prescribing in the primary care facilities visited. Where doctors were not prescribing paramedical staff or nurses generally prescribed, although in one country unqualified staff sometimes prescribed.
25	No prescribing by staff with less than 1 month’s training in public primary care	Policy was marked “yes” if no unqualified staff were observed to prescribe.

^a^Includes all the policy questions, hypothesised to act on the quality of medicines use, as hypothesised elsewhere [16, 17] and found in the situational analysis reports [21]

### Analyses

Analyses were done in Excel 2016 and Epi Info version 7.2.1.0. Univariate analyses, with each policy as the unit of analysis, were used to identify policies that were associated with better QUM. The mean difference (expressed as a percentage) for each QUM indicator between “countries” (country-year jurisdiction) implementing and not implementing a specific policy was calculated. The directionality of “better” or “worse” use was aligned for each of the six QUM indicators and an average (overall) difference calculated for each specific policy, whereby a positive (+) number indicates “better” use and a minus (−) number indicates “worse” use. The mean differences for each QUM indicator, and the average difference across all QUM indicators for each policy were calculated and represent an estimate of the quantitative impact of each policy. Head-to-head comparisons of the impact of different policies and further multiple comparisons were not done.

Correlations of multiple policies with QUM indicators, where the “country-year jurisdiction” was the unit of analysis, were performed to see if adoption (implementation or partial implementation) of more policies was associated with better QUM. Since various policies are likely to impact differently on different QUM indicators, and to gain an idea of the overall impact on QUM by the package of policies that any one country was implementing in one specific year, a composite QUM variable was derived, in the same manner as has been done elsewhere [16]. Use of a composite QUM variable allowed comparison of data across all 20 situational analyses rather than only 15, since some QUM indicators were not measured in five situational analyses. For each individual QUM variable, we calculated how far that country’s value (referred to as “country-year” – see text) lay above or below the mean value from all “country-years” and then converted this difference into standard deviation units. The average of the standard deviation unit increments across all six QUM indicators for each country-year was calculated and this was then regressed against the number of implemented policies that were associated with an effect size of more than 1% (22 out of 25 policies) as estimated from the univariate analysis.

Although countries implement each policy differently, the adoption of more policies is likely to reflect stronger intention to promote QUM, which may be reflected variously by different QUM indicators. Hence, individual QUM indicators were also regressed on the number of policies a country implemented. Since there may be correlation between the results of different situational analyses done in the same country, a sensitivity analysis was done by restricting the regression analysis to the latest situational analysis of the country.

The impact of country economic status was assessed by including Gross National Income per capita (GNIpc) into multiple linear regression analyses and by repeating the regression analyses for countries with GNIpc above and below the median of USD 2230.

Finally, in those countries where a situational analysis was done twice, the mean difference between situational analyses, for each of the six QUM indicators, was calculated to see whether there had been any change in QUM over time, and whether any change was accompanied by a corresponding change in the number of policies implemented.

## Results

Data were extracted from 20 situational analysis reports covering all 11 countries of the WHO South-east Asia region – two reports from eight countries in different years, two reports from India (one North Indian and one South Indian state in the same year) and one report each from two countries (Democratic Peoples’ Republic [DPR] of Korea and Indonesia). QUM data for all six indicators were extracted from 15 country-visit reports, with 1–2 QUM variables missing from four reports (concerning injection use, prescribing from the Essential Medicines List [EML] and antibiotic use in upper respiratory tract infection [URTI] cases), and 5 out of six QUM indicators missing from one early report (Bangladesh 2010). A total of 206 public-sector health facilities were visited (average of 10–11 public-sector health facilities per country-visit, half of which were primary healthcare centres) and 30 prescriptions per facility were examined to estimate five of the six QUM indicators. The QUM indicator concerning the % URTI cases treated with antibiotics was estimated from visits to 151 public-sector health facilities (average of 7–8 facilities per country visit) with an average of 23 URTI prescriptions examined per facility. In two countries (Sri Lanka 2010 and Bangladesh 2010) no data on antibiotic use in URTI was available, in two countries (Bhutan 2011 and Maldives 2011) data on antibiotic use in URTI was available in only two health facilities and in one country (Indonesia 2011) from only three health facilities. Policy data for all 25 indicators were extracted from all 20 country-visit reports.

### Comparison of QUM indicators in countries with and without specific policies

**Fig. 1:**
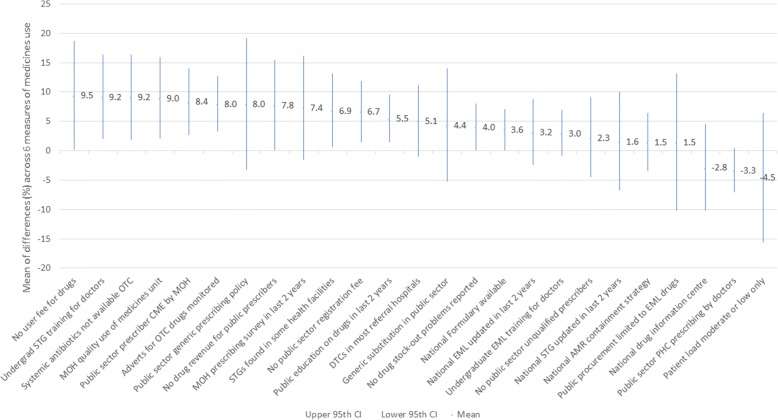
Differences in medicines use between countries with and without medicines policies. STG = Standard Treatment Guidelines; OTC = Over-the-Counter; MOH = Ministry of Health; CME = Continuing Medical Education; DTC = Drug and Therapeutic Committee; EML = Essential Medicines List; AMR = Antimicrobial Resistance; PHC = Primary Health Care

Table 4 shows the mean differences for each of the six QUM indicators, and the average difference across all six QUM indicators, between countries that did and did not implement the 25 policies hypothesised to be associated with better use. Figure 1 shows the mean difference (with 95% confidence interval)) across all six QUM indicators for each of the 25 policies. Twenty-two out of 25 policies were associated with better QUM, although in many cases the differences were small. Twelve policies were associated with statistically significantly (*p* < 0.05) better QUM of more than 3.6% – namely not charging patients any user fee or copayment for medicines at the point of care, undergraduate education of prescribers on the national treatment guidelines (STGs), distribution of STGs to health facilities (as demonstrated by finding them in facilities), a Ministry of Health (MOH) unit dedicated to promoting QUM, continuing education on prescribing for health workers by MOH, general non-availability of systemic antibiotics over-the-counter (OTC), monitoring of advertisements for OTC medicines, no revenue from medicine sales for public sector prescribers, having no stock-out problems, public education and having a national formulary. For the 22 (out of 25) policies associated with better overall QUM of more than 1%, 96 (73%) of a possible 132 comparisons (6 QUM indicators × 22 policies) were associated with better QUM.

**Table 4:** Differences in medicine use between countries with and without each of 25 policies hypothesised to be associated with better use

Policy	Number of countries with policy (out of 20)^a^	% URTI cases treated with antibiotics	% patients treated with antibiotics	% medicines prescribed from the EML	% medicines prescribed by generic name	% patients prescribed multivitamins	% of patients prescribed injections	Average % better (+) medicines use with policy (95% CI)
Direction of better medicines use: More (+); Less (−)		Less (−)	Less (−)	More (+)	More (+)	Less (−)	Less (−)	Sign changed where less use is better use
No user fee for drugs at most public health facilities	17	−9.6	+ 0.1	+ 9.5	+ 31.2	−7.5	+ 0.8	9.5* (0.2 to 18.7)
Undergraduate education of prescribers on STGs	5	−15.9	−5.2	+ 12.0	+ 22.0	− 1.1	+ 0.9	9.2* (2.1 to 16.4)
Systemic antibiotics mostly not available OTC	3	−16.0	− 5.2	+ 12.0	+ 22.0	− 1.3	+ 1.4	9.2* (1.9 to16.4)
MOH unit on Rational Use of Medicines established	3	−17.3	−10.9	+ 0.1	+ 19.7	+ 0.9	−6.9	9.0* (2.1 to 15.9)
Some public-sector prescriber CME by MOH	8	−7.5	−1.6	+ 5.7	+ 21.9	− 8.7	− 4.8	8.4* (2.7 to 14.0)
Advertisements for OTC drugs monitored	7	−14.5	−3.8	+ 4.0	+ 15.5	− 2.0	− 8.3	8.0* (3.4 to 12.7)
Public sector generic prescribing policy	9	−3.9	−1.6	+ 9.8	+ 35.1	+ 0.4	+ 2.2	8.0 (− 3.2 to 19.1)
No drug revenue for public sector prescribers	19	−19.1	−2.7	−2.2	+ 18.8	− 7.0	−6.7	7.8* (0.1 to 15.5)
MOH prescribing survey done in the last 2 years	7	−4.7	−1.2	+ 4.6	+ 28.6	+ 2.7	− 7.7	7.4 (− 1.4 to 16.2)
STGs found in some public health facilities	5	− 11.4	−9.7	+ 6.1	+ 17.8	+ 1.4	+ 2.4	6.9* (0.6 to 13.1)
No public-sector registration or consultation fee	12	−13.0	−3.3	+ 5.6	+ 15.2	+ 2.5	− 5.6	6.7* (1.5 to 11.9)
Some public education on medicines use in the last 2 years	5	−11.7	−7.4	+ 2.5	+ 8.9	−5.0	+ 2.4	5.5* (1.5 to 9.5)
DTCs in most public referral hospitals	8	−1.7	+ 3.2	−2.5	+ 15.2	−9.3	− 10.3	5.1 (− 0.9 to 11.2)
Generic substitution in the public sector	15	+ 6.0	+ 7.8	+ 16.1	+ 21.8	−2.8	+ 0.4	4.4 (−5.2 to 14.0)
No drug stock-out problems reported	9	−11.6	−3.8	−0.1	− 1.4	−2.3	− 7.9	4.5* (0.1 to 8.0)
National Formulary available	7	−8.7	−0.7	−3.4	+ 6.1	− 5.9	− 3.7	3.6* (0.2 to 7.1)
National EML updated in the last 2 years	12	−6.0	−0.7	+ 16.1	− 0.4	+ 1.2	+ 2.2	3.2 (− 2.4 to 8.7)
Undergraduate education of doctors on the EML	6	−9.9	+ 0.6	−3.5	+ 1.7	−3.7	−6.8	3.0 (−0.9 to 6.9)
No public-sector unqualified prescribers	18	−5.8	−6.5	+ 8.4	+ 9.8	+ 8.4	+ 8.6	2.3 (−4.5 to 9.0)
National STG updated in the last 2 years	7	+ 5.8	+ 6.9	+ 6.9	+ 20.1	+ 5.5	− 1.0	1.6 (−6.7 to 10.0)
National AMR Containment Strategy	4	−1.3	−3.7	−10.0	+ 2.2	− 3.4	− 8.3	1.5 (− 3.4 to 6.4)
Public procurement limited to EML drugs only (excl. DPRK)	15	+ 12.6	+ 12.5	+ 15.5	+ 22.3	+ 0.2	+ 3.4	1.5 (− 10.1 to 13.1)
National Drug Information Centre	2	+ 7.2	−1.5	−3.9	+ 11.6	+ 3.4	+ 15.7	− 2.8 (− 10.1 to 4.4)
Public sector PHC prescribing by doctors	13	+ 1.9	+ 3.5	−2.3	−10.3	−4.0	+ 5.8	−3.3 (− 7.1 to 0.4)
Prescriber patient load moderate or low (< 60 patients /prescriber/day)	12	− 10.9	−8.7	−5.4	−6.3	+ 27.2	+ 7.9	− 4.5 (− 15.5 to 16.5)

*p ≤ 0.05

^a^Sample size applies to the number of countries (out of 20) that had adopted the policy. The number of countries with and without policies for each individual QUM indicator varies slightly as certain QUM indicators were not measured in 5 country visits

*OTC* Over-the-counter, *STG* Standard treatment guidelines, *MOH* Ministry of health, *CME* Continuing medical education, *DTC* Drug and therapeutic committee, *EML* Essential medicines list, *AMR* Antimicrobial resistance, *DPRK* Democratic People’s Republic of Korea (which had no published EML)

Some policies had large effects on one particular QUM indicator, but not on overall use (as judged by six QUM indicators). Generic prescribing policies were associated with greater generic prescribing; implementing the national EML (as indicated by having an updated EML and limiting public procurement to EML drugs) was associated with greater prescribing of EML medicines. However, low to modest prescriber workload (as defined by seeing less than 60 patients per day) was not associated with better overall QUM, although it was associated with lower antibiotic use.

### Effects of multiple policies and national wealth

**Fig. 2:**
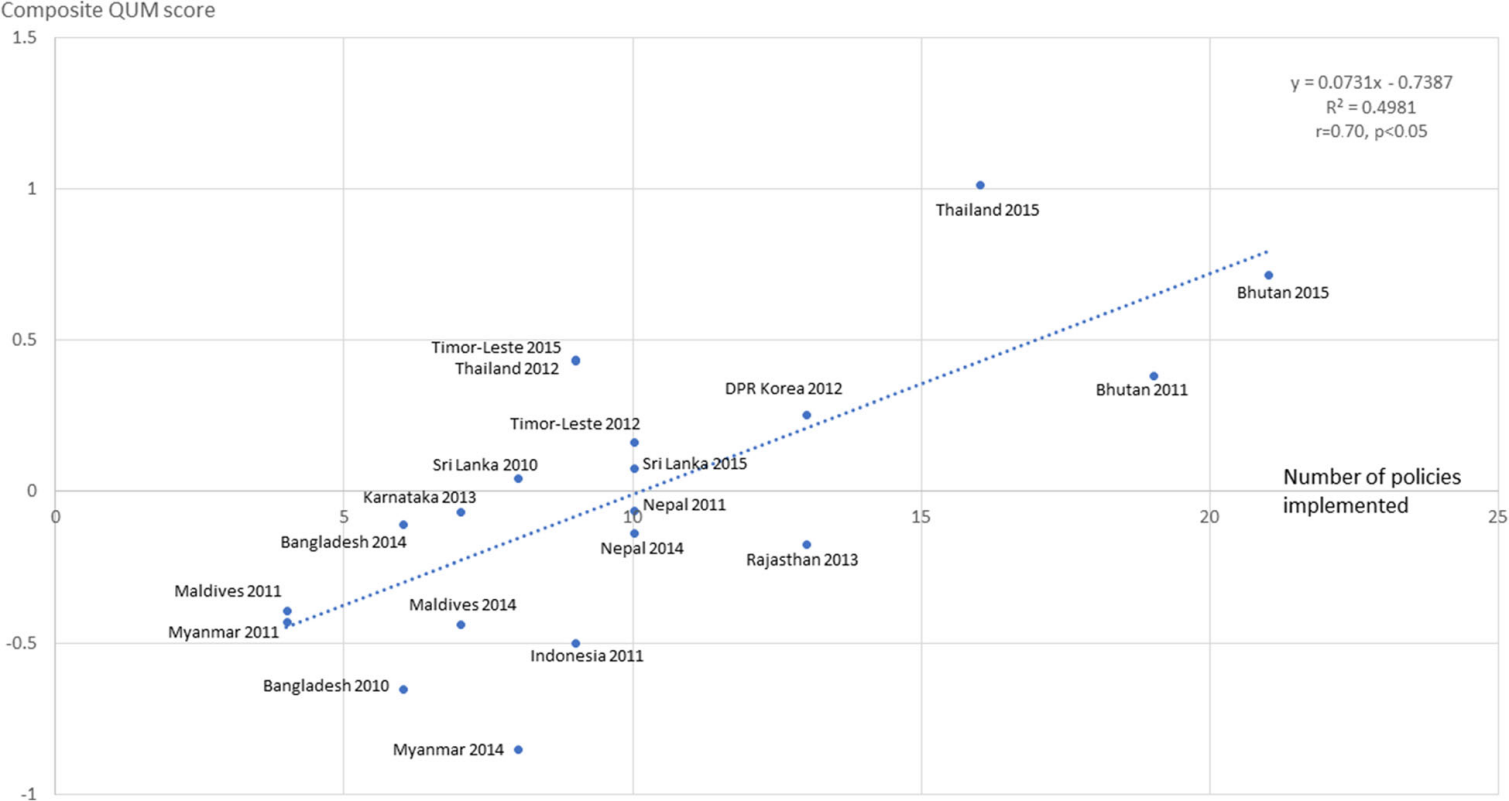
Scatter plot of composite QUM score versus number of policies (out of 22) implemented

Figure 2 shows a scatter plot of the composite QUM indicator and the number of policies implemented (out of 22 policies associated with better QUM) for 20 situational analysis visits (country-years). There was moderate to strong positive correlation between the number of essential medicines policies implemented and the composite QUM indicator (*r* = 0.71, r^2^ = 0.50, *p* < 0.05). In sensitivity analyses (not shown graphically) the correlation increased when GNIpc was included in the regression analysis (*r* = 0.75, r^2^ = 0.57, *p* < 0.05) and the correlation was moderate to strong in the 10 country-visits with GNIpc above USD 2230 (*r* = 0.76, r^2^ = 0.58, *p* < 0.05) and below USD 2230 (*r* = 0.69, r^2^ = 0.48, *p* < 0.05). Furthermore, confining the regression analysis to data from the most recent situational analyses in 12 states (all 11 countries including 2 Indian states), in order to take account of possible clustering of results from the same site, also showed moderate to strong correlation between the composite QUM indicator and the number of essential medicines policies (*r* = 0.71, r^2^ = 0.50, *p* < 0.05).

**Fig. 3:**
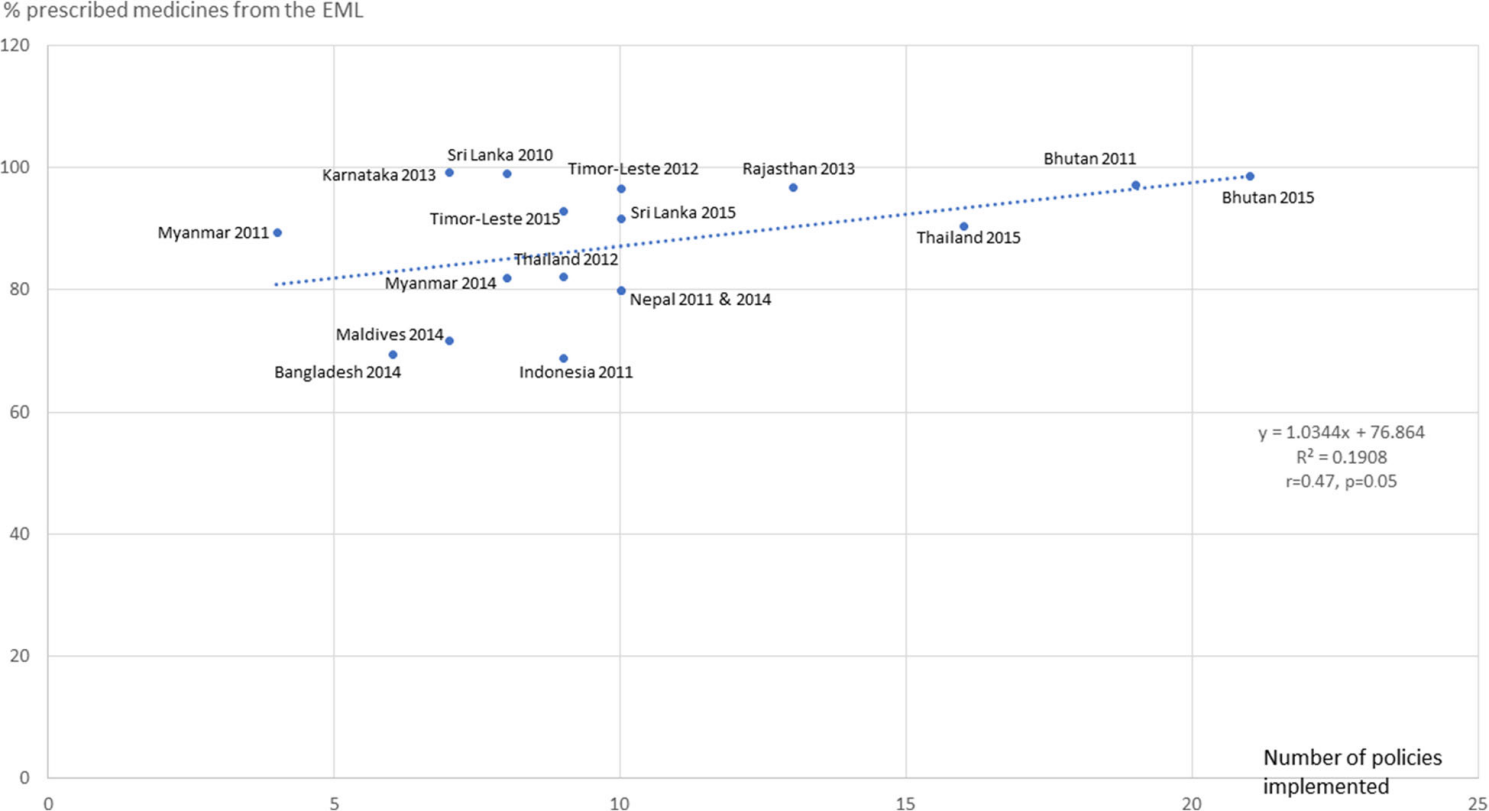
% prescribed medicines from the Essential Medicines List versus number of policies (out of 22) implemented

**Fig. 4:**
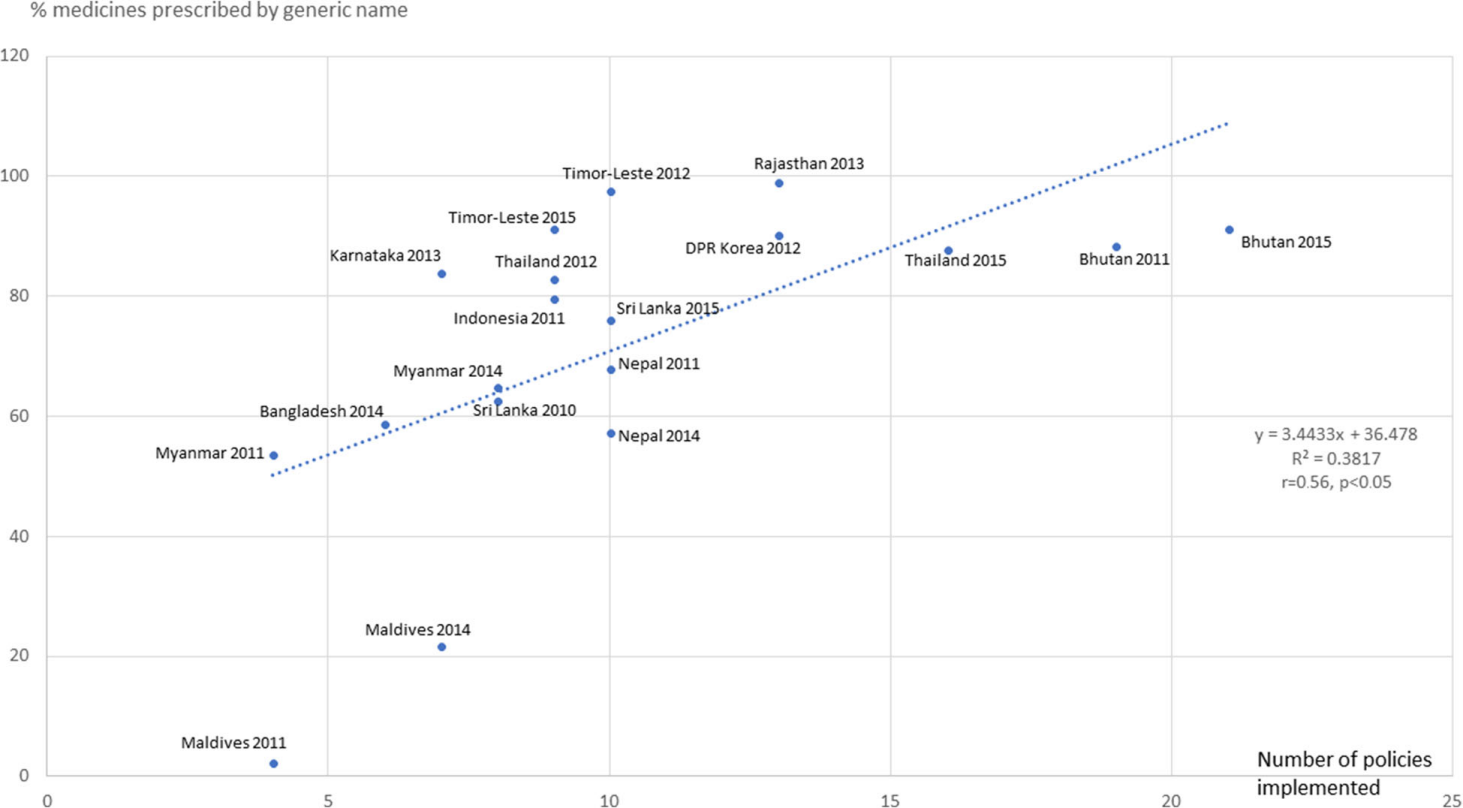
% medicines prescribed by generic name versus number of policies (out of 22) implemented

**Fig. 5:**
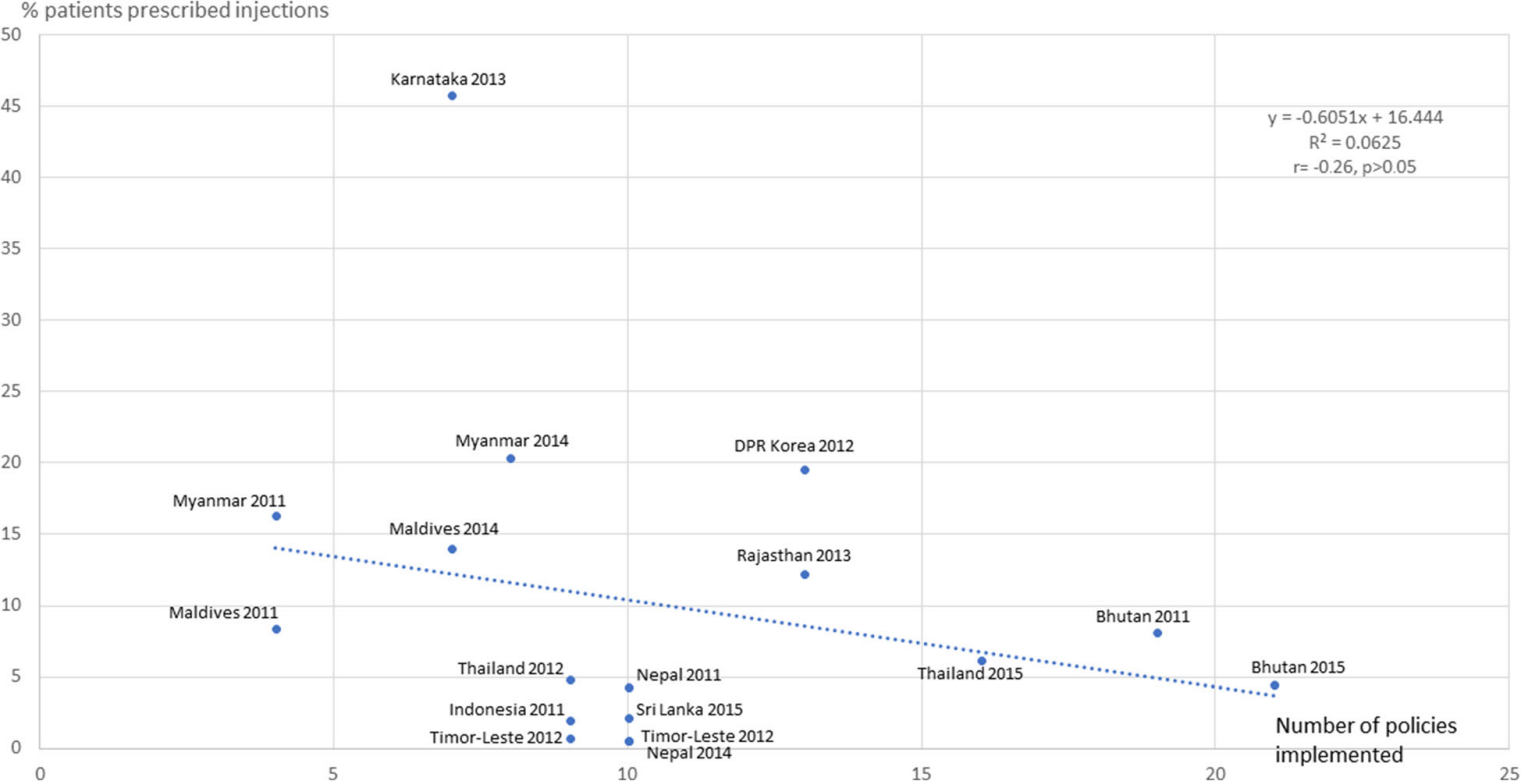
% patients prescribed injections versus number of policies (out of 22) implemented

**Fig. 6:**
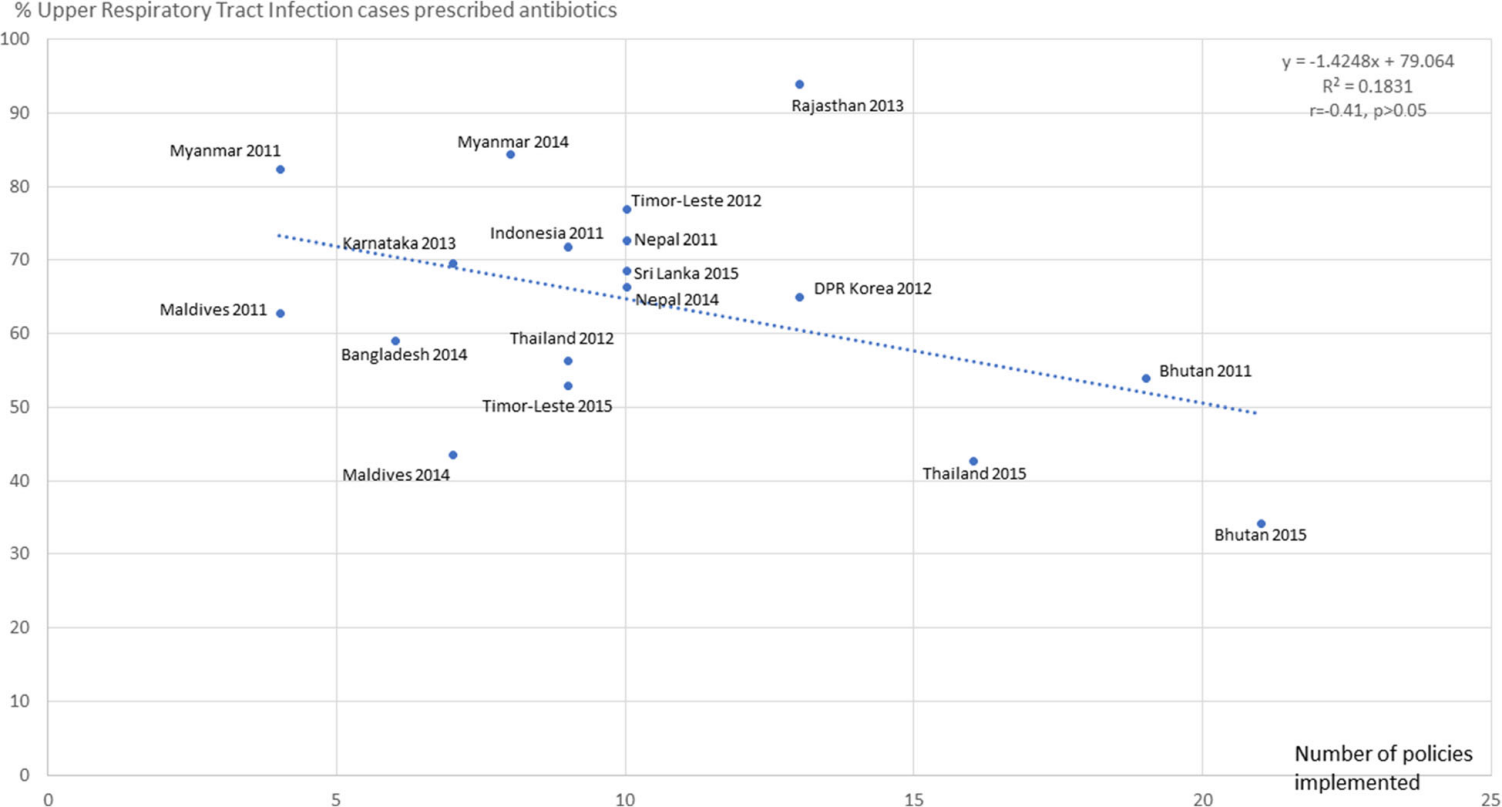
% Upper Respiratory Tract Infection cases prescribed antibiotics versus number of policies (out of 22) implemented

**Fig. 7:**
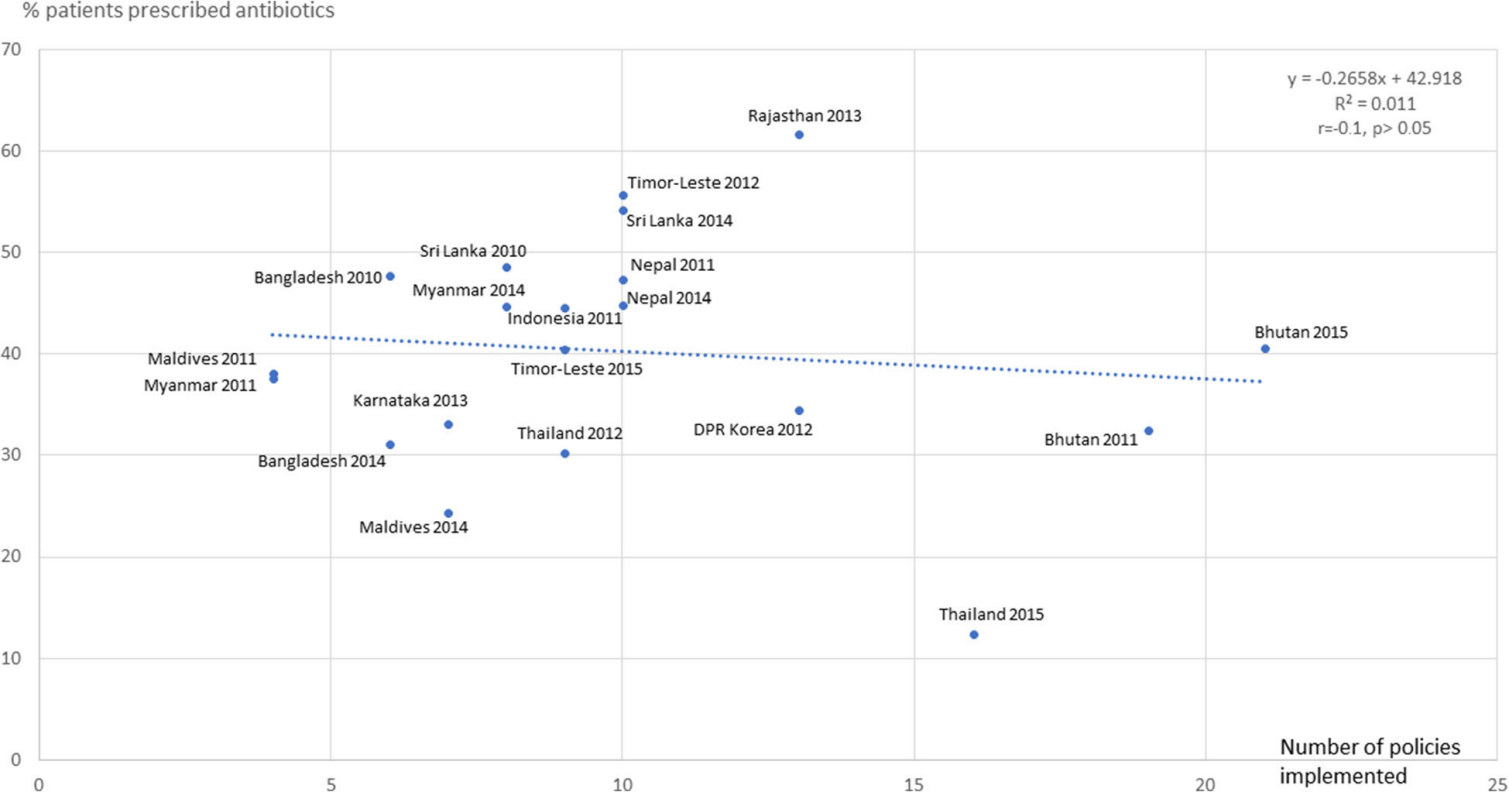
% patients prescribed antibiotics versus number of policies (out of 22) implemented

**Fig. 8:**
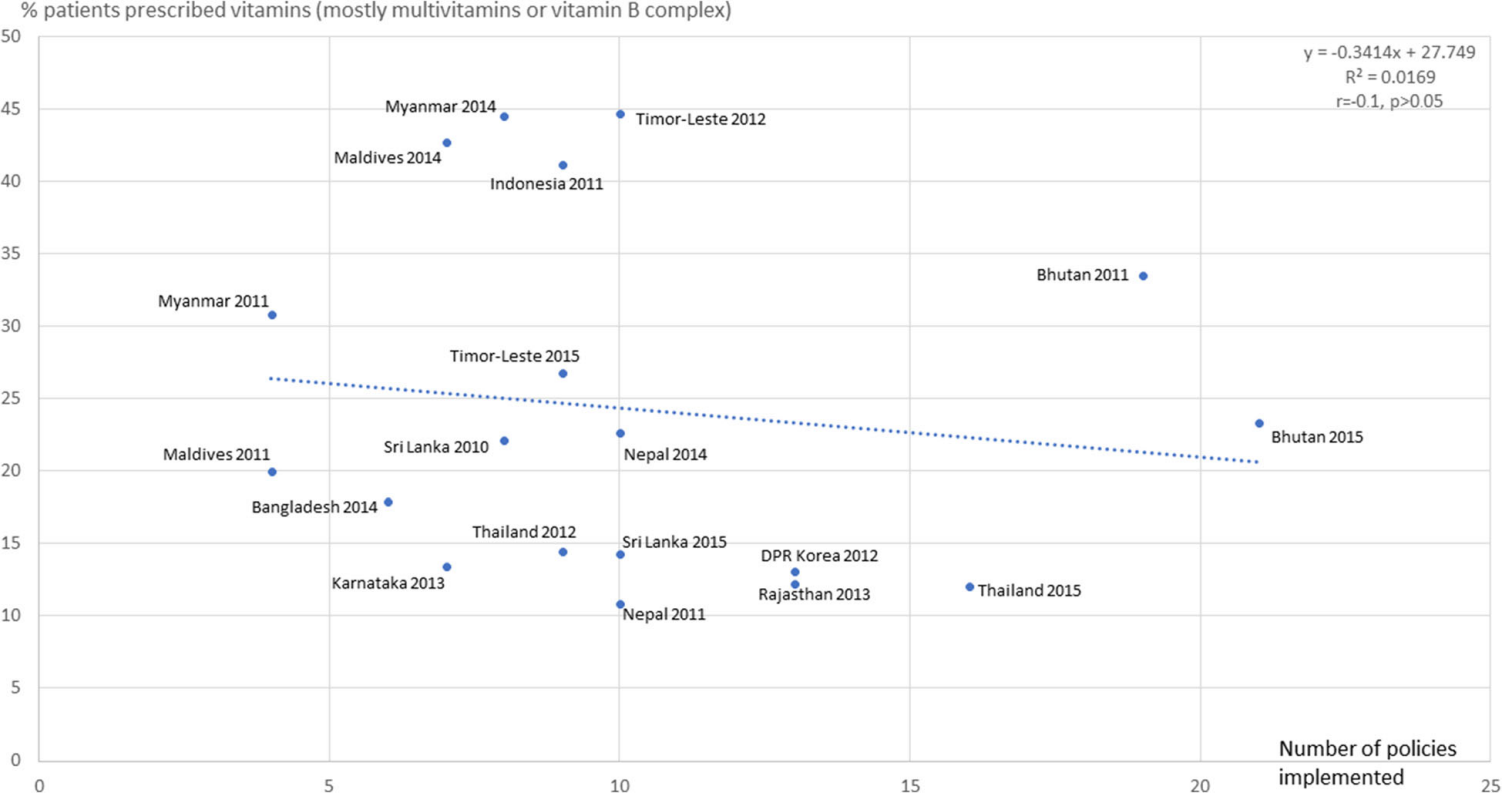
% patients prescribed vitamins versus number of policies (out of 22) implemented

Figures 3, 4, 5, 6, 7 and 8 show the scatter plots for the individual QUM indicators versus policy implementation. Regression analyses of individual QUM indicators and the number of policies (out of 22) implemented showed that with an increasing number of essential medicines policies implemented there was: an increase in the % prescribed medicines belonging to the EML (*r* = 0.46, r^2^ = 0.21, *p* > 0.05) (Fig. 3); an increase in the % medicines prescribed by generic name (*r* = 0.62, r^2^ = 0.38, *p* < 0.05) (Fig. 4); a decrease in the % patients prescribed injections (*r* = − 0.24, r^2^ = 0.06, p > 0.05) (Fig. 5) and a decrease in the % URTI cases prescribed antibiotics (*r* = − 0.42, r^2^ = 0.18, p > 0.05) (Fig. 6). There was virtually no change in the % patients treated with antibiotics (*r* = − 0.1, r^2^ = 0.01, *p* > 0.05) (Fig. 7) or vitamins (*r* = − 0.1, r^2^ = 0.02, *p* > 0.05) (Fig. 8) with increasing number of policies implemented. In a sensitivity analysis where policies not hypothesised to influence antibiotic use were excluded (2 policies on generic prescribing and substitution and 3 policies on EML implementation), a stronger association was found between the number of policies (out of 17) implemented and the % URTI cases prescribed antibiotics (*r* = − 0.49, r^2^ = 0.24, *p* < 0.05).

### Changes over time

In seven of the eight countries where a situational analysis had been done twice, separated by a period of 3–5 years, the average mean differences over time for the six individual QUM indicators and the average mean difference over the six QUM indicators, together with any change in the number of policies implemented are shown in Table 5. The data for Bangladesh were excluded because five of the six QUM indicators were missing from the first situational analysis in 2010. The data show a significant improvement in QUM together with increased policy implementation in Thailand, while in other countries there was no significant change in QUM and policy implementation. However, the small sample sizes (concerning health facilities and patient encounters) preclude further interpretation of the data.

**Table 5:** Changes over time in 8 countries where two country situational analyses were done

Country (Years of country situational analyses)	% URTI cases treated with antibiotics^a^	% patients treated with antibiotics^a^	% medicines prescribed from the EML^a^	% medicines prescribed by generic name^a^	% patients prescribed multivitamins^a^	% of patients prescribed injections^a^	Average % better (+) medicines use over time (95% CI)	Change in the number of policies implemented between the second and first situation analyses
Direction of better medicines use: More (+); Less (−)	Less (−)	Less (−)	More (+)	More (+)	Less (−)	Less (−)	Sign (+/−) changed for QUM indicators where less use is better use so that “+” = better QUM	
Bangladesh (2010, 2014)		−16.6						0
Bhutan (2011, 2015)	−19.6	+ 8.1	+ 1.5	+ 2.9	− 10.1	−3.7	+ 4.9 (−2.4 to + 12.5)	+ 2
Maldives (2011, 2014)	− 19.3	−13.8		+ 19.3	+ 22.8	+ 5.6	+ 4.8 (− 10.0 to + 19.6)	+ 3
Myanmar (2011, 2014)	+ 2.0	+ 7.1	−7.6	+ 11.3	+ 13.7	+ 4.0	− 3.8 (− 10.6 to + 2.9)	+ 4
Nepal (2011, 2014)	− 6.3	−2.5	−0.04	−10.5	+ 11.7	−3.7	− 1.6 (− 7.8 to + 4.5)	0
Sri Lanka (2010, 2015)		+ 7.1	− 7.5	+ 13.4	−7.8		+ 1.7 (− 6.8 to + 10.1)	+ 2
Thailand (2012, 2015)	−13.7	− 17.9	+ 8.4	+ 5.0	− 2.5	+ 1.3	+ 7.7 (+ 2.0 to + 13.4)**	+ 7
Timor-Leste (2012, 2015)	−24.1	−13.7	−3.7	−6.4	− 17.9	+ 0.1	+ 7.6 (− 2.5 to + 17.7)	−1

^a^The mean difference between the second and first situational analyses

**p ≤ 0.05

## Discussion

This study has two important findings. Firstly, some policies were associated with significantly better QUM; and secondly the more of these policies a country implemented the better was the QUM.

Policies statistically significantly associated with more than 7% better overall QUM were: not charging patients any user fee or co-payment for medicines at the point of care; implementation of STGs through undergraduate education of prescribers and adequate distribution (as indicated by finding STGs available at health facilities); an MOH unit dedicated to monitoring and promoting QUM; continuing medical education (CME) on prescribing for health workers by MOH; limiting the OTC availability of systemic antibiotics; not allowing public sector prescribers to gain revenue from the sales of medicines; and monitoring advertisements of OTC medicines. Other policies that had a significant but smaller association with better QUM included: more efficient drug supply system (as indicated by no stock-out problems reported); availability of a national formulary; public education programs on medicines use; and not charging patients any registration or consultation fee at health facilities.

The policies associated with better QUM in this study are similar to those policies associated with better QUM in an analysis of global secondary data [16, 17] (excluding those policies which were not commonly measured in both studies). The one exception was that CME was done by MOH, and was associated with better QUM in this study, but may not have been done by MOH in countries included in the previous analysis of global data, where CME was associated with poorer QUM.

The effectiveness of similar interventions has been reported elsewhere, including: education of prescribers [4, 5] and the public [26], an MOH unit dedicated to promoting QUM [27], administrative interventions such as hospital drug and therapeutic committees (DTCs) [28, 29], non-allowance of prescriber income from drug sales [30], non-allowance of antibiotics OTC [31], and monitoring of drug promotional activities [32]. As found in analysis of global data where provision of medicines free of charge was associated with better use [16, 17], so in this study not charging patients any fees for medicines (or fees that could be construed by patients as being for medicines e.g. registration and consultation fees) was associated with better use.

There was a significant moderate to strong positive correlation between the number of policies implemented and the composite QUM (over six indicators), and for two of the individual QUM indicators (% URTI cases prescribed antibiotics and % medicines prescribed by generic name). While the effect sizes were small for individual policies (< 10%), the effect sizes associated with implementation of multiple policies were large (30–95% over different QUM indicators) and comparable with the largest intervention effects reported elsewhere [4, 5]. We believe that the data on the possible impacts of multiple policies are important and likely to reflect a causal association. This is the second time we have found the correlations between numbers of implemented policies and better QUM measures, and these analyses were conducted in different and independent data-sets [16, 17].

Increased effect sizes with multiple (as opposed to single interventions) have been reported in many literature reviews [4, 5, 33–35]. The correlations between the composite QUM indicator and the number of policies implemented remained strong in both wealthier and poorer countries when the regression analysis was repeated for countries with a GNIpc above and below the median of USD 2230. This finding together with the fact that there was no correlation between GNIpc and the number of policies implemented suggests that it is likely that the better QUM seen with increasing policy implementation was due to the policies themselves and not due to wealth, as has been found elsewhere [16, 17].

The apparent improvement in QUM in association with increased policy implementation over time seen in Thailand may be a chance finding since the number of facilities was small and the same facilities were not visited during both visits. Nevertheless, Thailand increased its policy implementation more than other countries over the time-period and similar results have been reported elsewhere [36, 37]. Therefore, it is suggested that the method used in this study to measure QUM in relation to policy implementation may be a good method to monitor country progress on promoting QUM.

The data analysed in this study were collected from all countries during 2-week periods using a pre-designed workbook tool by government staff with facilitation from WHO, were discussed in each country and a report produced and published on WHO/SEARO website for future use [21]. The approach was mandated by Member States [38, 39] and proved relatively cheap and quick, so enabling government participation and action. This study further shows that the data collected from this approach is sufficient for regional analysis and development of a regional approach to promote better QUM.

### Limitations

Although there were moderate to strong correlations between medicines use and policy implementation in this study, causality cannot be proved and may be due to co-interventions. Small sample sizes disallowed multi-variable analyses. Nevertheless, these are the best data available on policy effectiveness in the public sector in South-East Asia given the absence in all countries of national longitudinal data which could be used to perform time series analysis to show association or possibly prove causality. It is acknowledged that much health care is provided in the private sector but there was insufficient data to investigate this. Nevertheless, the findings are very relevant since many prescribers work in both the public and private sectors and many private-sector prescribers copy public-sector ones e.g. private general practitioners copying public-sector specialists.

The policy data, recorded in the situational analysis country reports [21], was collected by direct observation and discussion with health officials and health facility staff during the country situational analysis visits and thus may be more accurate than data relying on MOH questionnaires, as used elsewhere [16, 17]. Nevertheless, there may be some misclassification since relatively few health facilities were visited and it was not always easy to interpret whether a policy was implemented or not. For example, there was enormous variation between countries and between health facilities in the same country concerning hospital DTC activities, and the content and extent of prescriber CME and public education provided by the MOH. Some policies measured in this study are likely to be effective through association with other health system factors and policies. For example, the general lack of availability of systemic antibiotics OTC in Bhutan and DPR Korea may be due to a lack of private sector as well as regulatory controls. The policy “no stock-outs reported” was assumed to reflect better drug supply which might then impact on QUM. However, a situation of “no stock-outs” could itself result from better QUM.

The prescribing (QUM) data were collected by direct examination of treatment in 30 outpatient patient encounters (plus 10–30 URTI cases where measured) per facility on the day of the visit using the INRUD/WHO methodology [23, 24]. Since all six QUM indicators were collected in 15 (75%) of the 20 country situational analysis visits, the QUM data may be more robust than what we used in other work, where we relied on previously published surveys with on average only three QUM indicators per country [16]. Nevertheless, while every effort was made to collect the data in a standard way, the variation between countries in documentation of patient treatment and the small sample sizes involved may have resulted in some inaccuracies in the results. Furthermore, some indicators such as injection use and antibiotic use in URTI cases could not be measured in some countries and in some health facilities within a country. However, any inaccuracies in the QUM and policy data would tend to weaken the correlations observed.

Another serious weakness is that the QUM measurements were based on small convenience samples (by MOH) of on average 10–11 public health facilities per country visit. Thus, the surveys were not generalisable to whole countries and no benchmarking of country performance can be done using these data. Nevertheless, implementation of some policies was judged by observation of what occurred in the health facilities where QUM surveys were done, and this may account for the stronger correlation between policy implementation and QUM seen in this study compared to previous analyses [16, 17].

## Conclusions

Irrational use of medicines is a serious problem in South-East Asia. Essential medicines policies were found to be associated with better QUM and it is recommended that all countries: implement (through adequate education and distribution) national STGs, EML and formulary; establish an MOH unit dedicated to QUM; conduct CME on prescribing for health workers under close supervision of MOH; limit OTC availability of systemic antibiotics; disallow public sector prescribers to gain revenue from the sales of medicines; monitor advertisements of OTC medicines; run public education campaigns on QUM; do not charge patients user fees or co-payments for medicines at the point of care; and invest in a more efficient drug supply system. The situational analysis approach allows the relatively quick and cheap collection of data on QUM and policy implementation which can be used to monitor progress and plan future action.

## Additional files

Additional file 1: Dataset created from extraction of data from country situational analysis reports, all available online at: http://www.searo.who.int/entity/medicines/country_situational_analysis/en/ and http://www.searo.who.int/entity/medicines/en/. Excel file of the dataset created from data extracted from the country situational analysis reports and which was used in the analysis described in this manuscript. (XLSX 13 kb)

Additional file 2: Medicines management in healthcare delivery: WHO/SEARO workbook tool and report template for undertaking a situational analysis of medicines management in health care delivery in low and middle-income countries, March 2016. Available online at: http://www.searo.who.int/entity/medicines/country_situational_analysis/en/. PDF file of the data collection instrument used to collect data in the country situational analyses and that was analysed in this manuscript. (PDF 1236 kb)

## Abbreviations

CME: Continuing medical education
DPR Korea: Democratic Peoples’ Republic of Korea
DTC: Drug and therapeutic committee
EM: Essential medicines
EML: Essential medicines list
GNIpc: Gross National Income per capita
INRUD: International Network for the Rational Use of Drugs
MOH: Ministry of Health
OTC: Over-the-counter
QUM: Quality use of medicines
SEARO: South-East Asia Regional Office
STG: Standard treatment guidelines
UN: United Nations
URTI: Upper respiratory tract infection
WHO: World Health Organisation
