# Supplementary material for: Promoting quality use of medicines in South-East Asia: reports from country situational analyses
Source: BMC Health Serv Res. 2018 Jul 5;18:526. doi: 10.1186/s12913-018-3333-1 (PMC6034320; doi:10.1186/s12913-018-3333-1)
Supplement: Supplementary file 2 — Medicines management in healthcare delivery: WHO/SEARO workbook tool and report template for undertaking a situational analysis of medicines management in health care delivery in low and middle-income countries, March 2016. Available online at: http://www.searo.who.int/entity/medicines/country_situational_analysis/en/. PDF file of the data collection instrument used to collect data in the country situational analyses and that was analysed in this manuscript. (PDF 1236 kb) [file 12913_2018_3333_MOESM2_ESM.pdf]

MEDICINES MANAGEMENT IN HEALTH CARE DELIVERY

Country Name:

Date of Situational Analysis:

WHO/SEARO workbook tool & report template for undertaking a situational analysis of medicines management in health care delivery in low and middle income countries

March 2016

INTRODUCTION AND INSTRUCTIONS

Aim of Country Situational Analysis of Medicines Management in Health Care Delivery

Effective management of medicines in health care delivery involves many functions, disciplines and stakeholders, covering the areas of supply, selection, use, regulation and policy. Generally, these functions are undertaken by a variety of different government units and other stakeholders. Often functions and fragmented due to poor coordination between these different government units and stakeholders. This, in turn, makes it difficult to plan strategically and effectively for the pharmaceutical sector. The aim of undertaking a country situational analysis of medicines in health care delivery is to gain a holistic understanding of how medicines are managed in the health care system, with respect to 5 main areas – supply, selection, use, regulation and policy. By understanding how medicines are managed in the health care system, one may identify the priority problems and solutions that are likely to be effective and feasible.

What does a country situational analysis involve?

The country situational analysis involves collection of both qualitative and quantitative data over a 2-week period and is followed immediately by a 1-day national workshop for all stakeholders. At the workshop, the findings of the situational analysis are presented and validated, group work is undertaken to identify the major priority problems and solutions and recommendations are agreed in plenary discussion. The recommendations are for use by MOH and partners in planning for the sector. The aim of the situational analysis is to identify a range of priority problems and solutions, not to collect sufficient data for generalizable estimates of function, which cannot be done in only 2 weeks. Even so the data that can be collected in 2 weeks (which is all most government staff can spare) is often surprisingly extensive and not available elsewhere. This instrument can used for reviewing management of traditional medicines also.

The situational analysis should involve visits to:

- *the major government departments concerned with medicines – medicines procurement & distribution, government health insurance (if there is significant population coverage), pharmacy services, medical services, drug regulation, drug policy, and any other department that is involved in medicines management and also any department of traditional medicine (if widely used in the country).*
- *the medical, nursing and pharmacy councils and associations (could be a joint meeting),*
- *any NGO/partner involved in medicines management*
- *two provinces (regions) and in each province a visit to 1-2 facilities of each type existing in the country e.g. 1 university hospital (medical school pharmacology department and the attached hospital); 1 district/provincial/regional public health office & drug warehouse; 2 district hospitals; 2 primary health care centres; 2 sub-centres/dispensaries; 2 -3 private pharmacies; and 1 public pharmacy (not attached to health facilities, if existing). This means altogether 16-24 facilities spread over 2 provinces (regions). If traditional medicine is widely used, 2 facilities dedicated to traditional medicine may be visited.*

Generally, where possible, in each situational analysis, new provinces/regions and new health facilities should be visited, not the same ones as were visited in the previous situational analysis. This is to ensure that over a period of years, the situational analysis covers as much as possible of the different geographical areas and is not limited to the most excellent facilities in the national capital. As mentioned previously, data collection is sufficient to elicit a range of problems, not conduct a generalizable survey to estimate national function.

At each facility, visits will be made to the following:

- *the health staff in charge of the facility to introduce the team and objectives and to ask general administrative details about the health facility;*
- *the main pharmacy or drug store to observe drug stock availability & to ask about drug supply;*
- *the outpatient pharmacy department to review of 30-60 prescriptions for general primary care type cases in the outpatients and to observe dispensing;*
- *the outpatient department (from prescriptions in the pharmacy or the patient registers) to review prescribing in 30 cases of uncomplicated upper respiratory tract infection*
- *the outpatient department to talk with the prescribers;*
- *1-2 inpatient wards to observe how medicines are managed and dispensed.*

The situational analysis should be done by a team of 4-8 government officials drawn from government bodies responsible drug supply, drug selection, drug use and quality of care, drug regulation, drug policy and health insurance. If traditional medicine is widely used in the country and there are public traditional medicines services then an additional government official from the department of traditional medicines may be invited to join the team. In addition, there should be a person in-country to coordinate the process and also an external international facilitator to supervise data collection and analysis and report writing by the government team, and to moderate discussion between different government bodies during the national workshop.

Since the main aim of the situational analysis is learn about the health system and not to name and shame people, it is important to treat all respondents with respect and treat all information provided by individuals confidentially i.e. not publish who said what.

How to use this workbook tool

This workbook tool is designed such that information concerning medicines supply, selection, use, regulation and policy may be entered into the relevant sections in a systematic manner. The core sections of the workbook on drug supply, selection, use, regulation and policy, sections 1- 12, will eventually form the report. The health facility, public health office/warehouse and retail pharmacy survey forms, one per health facility, sections 13-15, are designed to facilitate systematic data collection at each facility. The data so collected should be analysed for each facility, and then across facilities, and the results entered into the relevant section in the workbook tool/report. While the tool can accommodate direct manual or electronic data entry, each team member should also use a notebook for supplementary notes. If necessary, information on traditional medicine maybe entered into the relevant section of the workbook. The workbook is designed such that each section starts on an odd number page and can therefore be printed separately. Thus, different sections can be used separately and simultaneously by different government team members.

Once collection of information and entry into the workbook is completed, the workbook itself will form the final report. In each section, there are instructions on what information should be included and what analyses should be undertaken and all these instructions are in italic red font.

When all information has been entered into sections 1-12 of the workbook and the report is being finalized, the instructions in each section, these pages on introduction and instruction, and sections 13-16 on the facility survey forms and preparation should be deleted.

CONTENTS

Please change the page numbers according to report drafted using this tool. Page

1. Abbreviations 7

2. Executive Summary

2.1 Introduction 8
2.2 Medicines Supply 9
2.3 Medicines Selection 10
2.4 Medicines Use 11
2.5 Medicines Regulation 12
2.6 Medicines Policy 13

3. Programme 14

4. Medicine Supply 15

4.1 Responsible Agents/Departments 16
4.2 Drug availability 16
4.3 Annual aggregate data of medicines distribution/consumption 19
4.4 Drug procurement 23
4.5 Allocation of Budget for medicines in the public sector 26
4.6 Drug quantification in the public sector 26
4.7 Drug Distribution in the public sector 27
4.8 Patient Flow in the Health Facilities 29
4.9 Insurance 30
4.10 Drug Manufacturing 31
4.11 Drug management in the private sector 32
4.12 Summary status in medicines supply since last situational analysis 33
4.13 Medicines Supply: Recommendations 34

5. Medicines Selection 35

5.1 National Essential Medicines List (EML) 36
5.2 Other Medicine Lists 37
5.3 Development / updating of national EML 38
5.4 Implementation of the EML 39
5.5 Summary status in medicines selection since last situational analysis 41
5.6 Drug Selection: Recommendations 42

6. Medicines Use 43

6.1 Responsible Agents / Departments 44
6.2 Past prescription surveys of medicines use done in the last 10 years 45
6.3 Current prescribing practices 46
6.4 Dispensing Practices 49
6.5 Policies to promote rational use of medicines 52
6.5.1 Monitoring and supervision of prescribing / dispensing 52
6.5.2 Standard Treatment Guidelines (STGs) 53
6.5.3 National Formulary 54
6.5.4 Drug Information Centre 54
6.5.5 Independent drug information 55
6.5.6 Drug and Therapeutics Committees 55
6.5.7 Undergraduate education on medicine use 56
6.5.8 Continuing Medical Education and medicines use 57
6.5.9 Public Education on the safe and prudent use of medicines 58
6.5.10 Generic Policies 58
6.6 Summary status in medicine use since last situational analysis 59
6.7 Medicines Use: Recommendations 60

7. Medicines Regulation 61

7.1 Responsible Agents/Departments 62
7.2 Pharmaceutical sector 63
7.3 Current Medicines Legislation (key documentation) 64
7.4 National Regulatory Authority for medical products 65
7.5 Drug Schedules 68
7.6 Regulation and inspection of drug outlets 69
7.7 Drug Registration 70
7.8 Pharmacovigilance 71
7.9 Drug Promotion 72
7.10 Drug Price Controls 72
7.11 Drug Testing Laboratories 73
7.12 Drug recall 74
7.13 Clinical Trial Oversight 74
7.14 Licensing and Accreditation of Health Professionals 75
7.15 Licensing and Accreditation of Health Facilities and Pharmacies 76
7.16 Summary status in drug regulation since last situational analysis 77
7.17 Medicines regulation: Recommendations 78

8. Medicines Policy and Coordination 79

8.1 National Medicines Policy Documents 80
8.2 Summary of medicines policies in place to promote rational use of medicines 81
8.3 Coordination of medicines-related policies within Ministry of Health 82
8.4 Other Ministries with medicines-related functions 84
8.5 Summary status in medicines policy since last situational analysis 85
8.6 Medicines Policy & Coordination: Recommendations 86

9. References 87

10. Persons met during the situational analysis 88

11. Participants of the Stakeholder Workshop 89

12. Workshop Slide Presentation 90

13. Health Facility Survey Forms 91

14. Public Health Office/Warehouse Survey Forms 113

15. Retail Pharmacy Survey Forms 121

16. Preparation 129

16.1 Preliminary consultations 129
16.2 Distribution of this workbook tool 129
16.3 Authorization and approvals 129
16.4 Identification of key stakeholders and respondents 130
16.5 Budget 130
16.6 Assembly of assessment team 130
16.7 Arrangement for coordination and supervision 130
16.8 Identification and location of key literature 131
16.9 Arrangement for health facility surveys 131
16.10 Stakeholder workshop' 132
16.11 Situational Analysis Report 132
16.12 Confidentiality 132

1. ABBREVIATIONS

Please adapt according to locally used acronyms and abbreviations.

ABC	ABC analysis – method for measuring drug consumption
ADR	Adverse Drug Reaction
AMR	Antimicrobial Resistance
CME/CPD	Continuing Medical Education / Continuing Professional Development
DHO	District Health Office
DIC / MIC	Drug / Medicines Information Centre
DRA	Drug Regulatory Authority
DSO	Drug Supply Organisation
DTC	Drug and Therapeutics Committee
GDP	Good Dispensing Practice
EM/ED	Essential Drugs / Essential Medicines
EDL/EML	Essential Drug List / Essential Medicines List
GMP	Good Manufacturing Practice
GPP	Good Prescribing Practice
HOD	Head of Department
HP	Health Post
IPD	In-patient Department
M&E	Monitoring & Evaluation
MO	Medical Officer
MOH	Ministry of Health
NDP/NMP	National Drug Policy / National Medicines Policy
NF	National Formulary
NGO	Non-Governmental Organisation
OPD	Outpatient Department
OTC	Over-the-Counter
PBPT	Problem-based Pharmacotherapy
PHC	Primary Health Care
PV	Pharmacovigilance
QA	Quality Assurance
RUM	Rational Use of Medicines
SOP	Standard Operating Procedures
STG	Standard Treatment Guidelines
TOR	Terms of Reference
TRM	Traditional Medicines
VEN	Vital, Essential, Non-essential – method for classifying drug importance
WHO	World Health Organization

2. EXECUTIVE SUMMARY

2.1. Introduction

Please adapt as necessary.

A situational analysis was conducted in *[insert country name]* during *[insert dates]*. The Terms of Reference were to examine medicines in health care delivery with respect to medicines supply, selection, use, regulation and policy. It was agreed that the WHO/SEARO workbook tool would be used and that a team of government officials, led by the [name of government department], facilitated by WHO/SEARO, would conduct the situational analysis.

The team members consisted of:

[List the team members]

The programme involved meetings with all the major government departments and other stakeholders involved in the management of medicines and visits to health facilities in two regions. A detailed program can be seen in section 3. During the visits to public health facilities and private pharmacies, drug stores were visited to collect data on stock availability for *[fill in the number]* selected essential drugs and drug management, outpatient dispensaries were visited to do a prescription audit, wards were visited to review in-patient drug management, and staff were interviewed to identify health and health care factors affecting drug management.

A one-day national stakeholder workshop was held on *[insert dates]* where findings were discussed and recommendations developed. The participants list can be seen in section 12. The findings were presented on behalf of the team by Dr Holloway, WHO/SEARO. Group work was done by participants to develop recommendations in the areas of medicines supply, selection, use, regulation and policy.

The words “medicine” and “drug” are used interchangeably in this report.

2.2. Medicines Supply

Please copy the sections on summary status and recommendations from section 4 on medicines supply.

2.3. Medicines Selection

Please copy the sections on summary status and recommendations from section 5 on medicines selection.

2.4. Medicines use

Please copy the sections on summary status and recommendations from section 6 on medicines use.

2.5. Medicines Regulation

Please copy the sections on summary status and recommendations from section 7 on medicines regulation.

2.6. Medicines Policy and Coordination

Please copy the sections on summary status and recommendations from section 8 on medicines policy and coordination.

3. PROGRAMME AGENDA

Please fill in the places visited and the dates visited.

Day	Date	Time	Places visited
1		Am	
		Pm	
2		Am	
		Pm	
3		Am	
		Pm	
4		Am	
		Pm	
5		Am	
		Pm	
6		Am	
		Pm	
7		Am	
		Pm	
8		Am	
		Pm	
9		Am	
		Pm	
10		Am	
		Pm	
11		Am	
		Pm	
12		Am	
		Pm	
13		Am	
		Pm	
14		Am	
		Pm	
15		Am	
		Pm	
16		Am	Workshop
		Pm	Workshop

4. MEDICINE SUPPLY

4.1 Responsible Agents/Departments

After discussion with MOH officials, please tick whether MOH or another agency is responsible for various drug supply functions and write the name of the agency in the table below.

Function/ Organisation	MOH	Other Agency	Name of Agency/MOH Department
Selection			
Quantification			
Procurement			
Pricing			
Storage			
Distribution			
Monitoring & evaluation			

4.2. Drug availability

(1) Describe any drug availability surveys done in the last 5 years.

(2) Describe briefly end-user views on drug availability using information collected from central officials and staff at the health facilities visited.

(3) Choose approximately 30-40 essential medicines whose availability at health facilities you are going to check. Ideally the list should include about 20-30 drugs that should be available at primary care and 10 drugs that should only be available only in hospitals. Once you have chosen the list of essential medicines, type these into a stock availability table for use in the survey forms – tables 13.5.8 (health facility), 14.5.10 (public health office/drug warehouse) and 15.2.1 (retail pharmacy) - and print out enough copies of the list so that one can be used in every health facility to be visited.

(4) Describe the methodology for the assessment of drug availability and stock-out undertaken during the health facility survey and include in the text the list the 30-40 essential medicines chosen by the team to investigate the % of key essential medicines available.

(5) Insert the results from each health facility survey on stock availability and stock-out into table 4.2.1

(6) Once the report is finalized by the government team and WHO, all health facility names in table 4.2.1 should be replaced by numbers (e.g. hospital 1, 2, 3, etc.) in order to maintain anonymity of individual health facility results.

(7) *The list should be chosen by the government team but should contain the following medicines as well as others that they might choose (bracketed drug names are examples only):*

a. *Tab/capsules:*
   i. *amoxicillin*
   ii. *2-3 non-penicilin antibiotics (fluoroquinolone, cephalosporin or macrolide);*
   iii. *antihelminthic (albendazole or mebendazole);*
   iv. *metronidazole;*
   v. *oral rehydration solution;*
   vi. *paracetamol and one other analgesic;*
   vii. *antihistamine;*
   viii. *iron and folic acid;*
   ix. *beta-blocker (atenolol);*
   x. *ACE inhibitor (enalapril);*
   xi. *diuretic (furosemide, thiazide);*
   xii. *metformin;*
   xiii. *sulphonyl urea (glibenclamide);*
   xiv. *H2 blocker (ranitidine) or proton pump inhibitor(omeprazole);*
   xv. *anti-depressant (amitriptyline)*
   xvi. *anticonvulsant (phenytoin or carbamazepine)*

b. *Injections/infusions:*
   i. *steroid (dexamethasone),*
   ii. *normal saline and/or ringer lactate;*
   iii. *analgesic (diclofenac);*
   iv. *cephalosporin (ceftriaxone)*
   v. *one non-penicillin antibiotic (carbapenem, gentamicin);*
   vi. *anticonvulsant (diazepam)*

c. *Respiratory solution:*
   i. *salbutamol,*
   ii. *steroid inhaler*

d. *Skin:*
   i. *Anti-scabies lotion (Benzyl benzoate or gamma benzene hexachloride);*
   ii. *Antifungal cream (clotrimazole or miconazole)*

e. *Eyes/ears:*
   i. *antibiotic drops*

Table 4.2.1: Summary of national EML drug availability from observation and record review in the health facility surveys:

Public Referral Hospitals	*Insert Name*	*Insert Name*	*Insert Name*	*Insert Name*	Average
% EML/currently used items out of stock*					
% key EML drugs available					
% prescribed drugs dispensed**					
Public District Hospitals	*Insert Name*	*Insert Name*	*Insert Name*	*Insert Name*	Average
% EML/currently used items out of stock					
% key EML drugs available					
% prescribed drugs dispensed**					
Public primary health care centre	*Insert Name*	*Insert Name*	*Insert Name*	*Insert Name*	Average
% EML/currently used items out of stock*					
% key EML drugs available					
% prescribed drugs dispensed**					
Private pharmacies	*Insert Name*	*Insert Name*	*Insert Name*	*Insert Name*	Average
% EML/currently used items out of stock*					
% key EML drugs available					
% prescribed drugs dispensed**					
Other facility types*** *Insert type*	*Insert Name*	*Insert Name*	*Insert Name*	*Insert Name*	Average
% EML/currently used items out of stock**					
% key EML drugs available					
% prescribed drugs dispensed**					

Please adapt the legend.
* *For the out-of-stock indicator, the team must choose whether the % EML items or the % of currently used items out of stock is used. In some countries, some EML items may not be supplied or used at the primary care level. In other countries with decentralized systems, local policy may not be to follow the national EML. In these circumstances it may be appropriate to measure the % currently used items out of stock. If this is done please record the numerator (number of products available) and the denominator (number of products regularly used).*
** From prescription audit done during the health facility survey
*** e.g. private hospital, private clinic, public pharmacy, outreach clinic

4.3 Annual aggregate data of medicines distribution / consumption

(1) Retrieve aggregate data on medicines consumption in the public sector by monetary value in the last fiscal year and undertake an ABC analysis to identify the top 20 medicines consumed by monetary value.

- *Public sector annual data may be found from the central government department responsible for medicines supply (for national level) and from the concerned government departments in those regions, provinces and districts visited during the situational analysis.*
- *If there is no one central government supply system of medicines (e.g. decentralized systems or systems relying on private supply), the ABC analysis should be done for the largest suppliers willing/able to provide data e.g. provincial/district government warehouses/stores, private importers, etc.*

(2) Retrieve aggregate data on medicines consumption in referral hospitals by monetary value in the last fiscal year and undertake an ABC analysis to identify the top 20 medicines consumed by monetary value.

- *Hospital annual data may be found from the pharmacy departments of large hospitals, whether public or private.*

(3) Describe what analysis you have done and the source of data, using the tabular format below.

- *Distribution or procurement data may be used.*
- *You may need several tables to describe consumption in several districts and hospitals but you should use one table to describe national level data. For districts and hospitals, you may present several districts in one table (side-by-side) or several hospitals in one table (side-by-side), but do not mix districts and hospitals in the same table.*
- *Please list all medicines in each table in descending order by value, starting with the medicine with the highest consumption by value.*

(4) After filling in all the tables of aggregate drug consumption, list the top 10 diseases and the top ten causes of mortality, referencing the sources, and comment on whether the expenditures on the top 20 drugs in the various ABC analyses are likely to be appropriate or not, bearing in mind the morbidity and mortality patterns.

Table(s) 4.3.1, 4.3.2 and 4.3.2 show the top 20 items consumed by value at national, district and hospital level respectively.

Table 4.3.1: ABC analysis of top 20 items – national level

Source of data (government department/organization): Year:

Rank	Item Name (including strength & formulation)	Unit costs	Monetary Value	EML Yes/No
1				
2				
3				
4				
5				
6				
7				
8				
9				
10				
11				
12				
13				
14				
15				
16				
17				
18				
19				
20				
	% budget on top 20 medicines:			
	% budget spent on antibiotics:			
	% budget spent on vitamins:			
	% budget spent on EML medicines:			
	% budget supplied centrally:			
	Per capita annual expenditure on medicines:			

If possible, comment briefly on how the unit prices compare within the Management Sciences for Health International Reference Prices.

Table 4.3.2: ABC analysis of top 20 items – district level

Source of data (government department/organization): Year:

Rank	District/Province 1			District/Province 2		
	Item Name/Strength	Monetary Value	EML	Item Name/Strength	Monetary Value	EML
1						
2						
3						
4						
5						
6						
7						
8						
9						
10						
11						
12						
13						
14						
15						
16						
17						
18						
19						
20						
	% budget on top 20 drugs			% budget on top 20 drugs		
	% on ABs			% on ABs		
	% budget on vits			% budget on vits		
	% budget on EML drugs			% budget on EML drugs		
	% value of drugs supplied centrally			% value of drugs supplied centrally		
	Per capita annual expenditure on medicines supplied			Per capita annual expenditure on medicines supplied		

Table 4.3.3: ABC analysis of top 20 items –hospital level

Source of data (government department/organization/hospital): Year:

Rank	Referral Hospital 1			Referral Hospital 2		
	Item Name/Strength	Monetary Value	EML	Item Name/Strength	Monetary Value	EML
1						
2						
3						
4						
5						
6						
7						
8						
9						
10						
11						
12						
13						
14						
15						
16						
17						
18						
19						
20						
	% budget on top 20 drugs			% budget on top 20 drugs		
	% on ABs			% on ABs		
	% budget on vits			% budget on vits		
	% budget on EML drugs			% budget on EML drugs		
	% value of drugs supplied centrally			% value of drugs supplied centrally		

4.4. Drug Procurement

Please describe drug procurement from review of reports and interview with staff of procurement agencies at the centre and periphery, remembering to include the following points:

- *Name of procurement agency?*
- *Is the procurement agency public/semi-autonomous/insurance/private?*
- *Is there procurement legislation?*
- *Is tendering (electronic or otherwise) undertaken?*
- *Who are the major suppliers and what supplier and product criteria are used?*
- *What medicines are procured - essential and non-essential medicines?*
- *Is procurement local and/or central?*
- *What is the lead time and what is the frequency of emergency orders?*
- *Are government funds provided in a timely manner?*
- *How is quality assured in the procurement process?*

4.4.1. National Public Sector Drug Procurement

4.4.2. Provincial/District/Health facility Drug Procurement

Table 4.4.1: Unit price comparisons between central and local purchases

If there is local procurement, please insert local and central prices in table 4.4.1 and calculate the % difference for those medicines where purchase has been done at the central and local levels. If several unit prices exist for a product from one source (central or local) take the average for the unit price. We are interested in how much greater local unit prices are than the central unit price so the calculation is: [(Local Unit Price – Central Unit Price) / Central Unit Price] x 100%.

Drug Name (include formulation & strength)	Central unit price (CUP)	Local unit price (LUP)	% difference
Average % difference [(LUP-CUP)/LUP] x 100%			

Table 4.4.2: Unit price comparisons between national central procurement and the Management Sciences for Health International Price Indicator Guide

For the top 20 medicines consumed by value and for the essential medicines selected for availability, please insert national central procurement unit prices and also unit prices for the equivalent products from the MSH international price indicator guide in table 4.4.2. Calculate the % difference between government and MSH unit prices. If several unit prices exist for a product from the national level take the average for the unit price. We are interested in how much greater national unit prices are compared to the MSH unit prices: [(National Unit Price – MSH Unit Price) / National Unit Price] x 100%.

Drug Name (include formulation & strength)	National unit price (NUP)	MSH unit price (MSHUP)	% difference
Average % difference [(NUP-MSHUP)/NUP] x 100%			

4.5. Allocation of budget for medicines in the public sector

Please describe allocation of budget from review of reports and interview with government staff at the centre and periphery, remembering to include the following points:

- *How is the budget allocated - on the basis of population? Number of beds in hospitals?*
- *What is the formula for allocating resources to provinces? Districts? Health facilities?*

4.6. Drug quantification in the public sector

Please describe drug quantification from review of reports and interview with government staff at the centre and periphery, remembering to include the following points:

- *How are quantities needed estimated? Past consumption? Morbidity?*
- *What is the formula (if any) for estimating quantities and is buffer stock included?*
- *Is actual estimation done according to the chosen method/formula (if any)?*

4.7. Drug Management and Distribution in the public sector

Please describe drug distribution from review of reports and interview with government staff at the centre and periphery, remembering to include the following points:

- *Name of distribution agency?*
- *Is the distribution agency public/semi-autonomous/insurance/private?*
- *Is there an electronic drug logistic management information system operating?*
- *Is the system a "push" or "pull" system?*
- *What is the time-table of drug orders/delivery?*
- *Is staffing sufficient?*
- *Are peripheral orders appropriate?*
- *Are drug deliveries according to orders?*
- *How many emergency orders were made in the last week/month/year?*
- *What are the problems*
- *Are traditional medicines supplied? If yes, please describe using the above bullets.*

4.7.1. Drug Storage and Distribution at the central national level

4.7.2. Drug Storage and distribution at the Provincial / District level (including redistribution)

4.7.3. Pharmaceutical Human Resources

Please describe what human resources are employed in drug supply management from review of reports and interview with government staff at the centre and periphery, remembering to include the following points:

- *Number of posts for pharmacists at central and provincial/district levels in drug supply management – for procurement, stock management and distribution*
- *Number of posts for non-technical staff at central and provincial/district levels in drug supply management – for procurement, stock management and distribution*
- *What proportion of the posts are filled?*

4.7.4. Traditional Medicine

Please describe whether traditional practitioners work in the same facilities as other government health care workers and whether traditional medicines are prescribed and supplied within the facility or whether patients must buy their traditional medicine products from outside shops. If traditional medicines are supplied to the health facilities, please describe the supply system.

4.8. Patient Flow in the Health Facilities (management of patient crowds)

Please describe how patient flow (patient crowds) is managed from observation at health facilities and interview with health facility staff, remembering to include the following points:

- *Patient Registration;*
- *Patient Triaging (screening) and direction to outpatients, emergency department, & inpatient wards;*
- *Patient fees for registration, diagnostics, medicines, inpatient beds;*
- *Patient flow from outpatient clinics to outpatient pharmacy;*
- *Record keeping;*
- *Number of patients treated daily in outpatients and inpatients;*
- *Are traditional medicines services are offered and if so, number of patients treated daily?*

4.9. Insurance

Please describe any insurance system from review of reports and interview with government staff at the centre and periphery, remembering to include the following points:

- *Names of insurance agencies;*
- *Public/private?*
- *Premiums?*
- *Benefit packages?*
- *Population coverage?*
- *Medicines covered?*
- *EML List/Reimbursement list?*
- *Reimbursement mechanisms*
- *Traditional medicine covered?*

4.10. Drug Manufacturing

Please describe drug manufacturing from review of reports and interview with central government staff, remembering to include the following points:

- *How many government-owned manufacturers?*
- *How many privately owned manufacturers?*
- *Traditional medicine manufacturers?*
- *Proportion of public-sector procured drugs from government-owned manufacturers?*
- *How is quality assurance maintained?*
- *What is the perceived quality by end-users?*

4.11. Drug Management in the private sector

Please describe how drugs were managed in the private pharmacies visited during the health facility survey, remembering the following points:

- *Situation of private pharmacies visited and the type of customers served e.g. near hospitals serving in-patients, or far away from hospitals serving private practitioners ;*
- *Type of pharmacies – whether owned by the pharmacist or by a chain;*
- *Qualification of staff serving customers;*
- *Approximate number of customers per day;*
- *Approximate value of sales per day;*
- *Approximate proportion of customers with prescriptions;*
- *Frequency of orders and number of suppliers;*
- *Dispensing practices*
- *Are traditional medicines dispensed in the same pharmacies as allopathic medicine? If yes, please describe the situation using the above bullets.*

4.12. **Summary status including progress, changes and problems** in drug supply since the last situational analysis

Have the recommendations from the last situational analysis been acted upon and, if not, why not?

4.13. Medicines Supply: Recommendations

5. MEDICINE SELECTION

5.1. National Essential Medicines List (EML)

Box 5.1. summarises the national EML

Please describe your review of the national EML in Box 5.1:

Box 5.1: Summary of National Essential Medicines List

- Responsible government department or agency:
- Date of publication of latest EML:
- Previous publication dates:
- Number of active pharmaceutical ingredients (APIs):
- Number of formulations for all APIs:
- Number of traditional medicine products:
- Categories by level of use:
    - Essential and complementary?
    - Facility type?
- Number of persons involved in drafting the latest EML:
    - Core team:
    - Experts:
    - Advisory Committee:
- Specialties represented:
    - Major specialties
    - General practice?
- Geographic representation of experts?
- Consistency with national STGs?

5.2. <u>Other Medicine Lists</u>

Please describe other medicines lists from record review and interview with government staff at the centre and periphery.

5.2.1. Central level

5.2.2. Province/District

5.2.3. Hospital

5.2.4. Insurance

5.2.5. Other

5.3. Development / updating of national EML

Please describe the development or updating process of the last national EML from interview with EML Committee Members and Chairperson, remembering to include the following points:

- *EML committee membership;*
- *Advisory experts;*
- *Selection criteria;*
- *Sources of evidence;*
- *Process for addition and deletions to EML;*
- *Transparency of the process;*
- *Conflict of interest;*
- *Budget.*

5.4. Implementation of EML

(1) Please describe implementation of the national EML from interview of the EML Committee Members and Chairperson, interview of government staff at the centre and periphery and observation at the health facilities visited, remembering to include the following points:

- *National policy with regard to EML implementation;*
- *Previous surveys on implementation of the EML;*
- *Availability of the EML in health facilities and on web;*
- *Use of EML in procurement;*
- *Training of providers on the EML;*
- *Consistency with national STGs;*
- *Views of prescribers on the relevance and usefulness of the EML.*

(2) Insert the results from the health facility survey on EML implementation into table 5.4.1.

(3) Once the report is finalized by the government team and WHO, all health facility names in table 5.4.1 should be replaced by numbers (e.g. hospital 1, 2, 3, etc.) in order to maintain anonymity of individual health facility results

Table 5.4.1 show some data on EML implementation.

Table 5.4.1: EML drug availability and use from observation and record review in the health facility surveys

Public Referral Hospitals	*Insert Name*	*Insert Name*	*Insert Name*	*Insert Name*	Average
% key EML items available*					
% items on supply list that are non-EML					
% prescribed drugs belonging to the EML**					
EML booklet available in pharmacy? Yes/No					
Public District Hospitals	*Insert Name*	*Insert Name*	*Insert Name*	*Insert Name*	Average
% key EML items available*					
% items on supply list that are non-EML					
% prescribed drugs belonging to the EML**					
EML booklet available in pharmacy? Yes/No					
Public primary health care centre	*Insert Name*	*Insert Name*	*Insert Name*	*Insert Name*	Average
% key EML items available*					
% items on supply list that are non-EML					
% prescribed drugs belonging to the EML**					
EML booklet available in pharmacy? Yes/No					
Private pharmacies	*Insert Name*	*Insert Name*	*Insert Name*	*Insert Name*	Average
% key EML items available*					
% items on supply list that are non-EML					
% prescribed drugs belonging to the EML**					
EML booklet available in pharmacy? Yes/No					
Other facility types*** *Insert type*	*Insert Name*	*Insert Name*	*Insert Name*	*Insert Name*	Average
% key EML items available*					
% items on supply list that are non-EML					
% prescribed drugs belonging to the EML**					
EML available in pharmacy? Yes/No					

* Belonging to the national EML or the provincial / hospital formulary in decentralized systems – please see the same indicator recorded in table 4.2.1.

** From prescription audit done during the health facility surveys – please see the same indicator recorded in table 6.3.1.

*** e.g. private hospital, private clinic, public pharmacy, outreach clinic.

5.5. **Summary status including progress, changes and problems** in drug selection since last situational analysis

Have the recommendations from the last situational analysis been acted upon and, if not, why not?

5.6. Drug Selection: Recommendations

6. MEDICINE USE

6.1. <u>Responsible Agents/Departments</u>

After discussion with MOH officials, please tick whether MOH or another agency is responsible for various functions to promote rational use of medicines (even if these functions are not undertaken) and write the name of the agency in the table below.

Function/ Organisation	MOH	Other Agency	Name of Agency/MOH Department
Monitoring medicines use in hospitals			
Monitoring medicines use in Primary care			
Development of national STGs			
Development of national formulary			
Drug Information Centre			
Provision of independent drug information			
Monitoring Hospital DTCs			
Monitoring Hospital quality of care			
Monitoring DTCs in provinces/districts			
Undergraduate education for health professionals			
Continuing medical education for health professionals			
Public education on medicines use			
Implementing generic policies			
Other functions *[specify]*			

6.2. Past prescription surveys

Please describe previous prescription surveys done in the last 10 years and fill in table 6.2.1 or an adaption of table 6.2.1.

Table 6.2.1 shows a summary of past reports of medicines use surveys in the last ten years.

Table 6.2.1: Reports of medicines use surveys done in the last 10 years

Indicators	Ref 1	Ref 2	Ref 3	Ref 4	Ref 5	Ref 6
Year of survey*						
Facility type**						
Public / private						
Average number of drugs per patient						
% patients prescribed antibiotics						
% patients prescribed injections						
% drugs prescribed by generic name						
% prescribed drugs belonging to the EML						
% patients prescribed vitamins						
% URTI patients prescribed antibiotics						
% pneumonia cases prescribed correct AB						
% diarrhoea cases treated with ORS						
% diarrhoea cases treated with AB						
Average cost per prescription (USD)						
% patients treated in compliance with STGs						
% patients treated with traditional medicines						
***						

* Year of survey refers to the year the survey was done not the publication date of the report;
** Referral hospital, district hospital, primary care centre; *** For other indicators of country's choice; AB=antibiotics; URTI=Upper respiratory tract infection; EML=essential medicines list; STG=Standard Treatment Guidelines.

6.3. Current prescribing practices

(1) Describe the methodology of the prescribing survey done during the health facility visits through prescription review, interview and observation, remembering to include the following points:

- *Location of data collection (e.g. outpatient department, dispensary, private pharmacy);*
- *Source of data (e.g. OPD patient register, dispensing register, prescriptions received in the pharmacy, patient records);*
- *Information available in the different sources of data (e.g. diagnosis, drug names, dosing schedules);*
- *Sample sizes in the different facilities.*

(2) Insert the results from each health facility survey prescription survey (see table 13.7.2 in the health facility survey form and table 15.3.5 in the retail pharmacy survey form) into table 6.3.2.

(3) Once the report is finalized by the government team and WHO, all health facility names in table 6.3.2 should be replaced by numbers (e.g. hospital 1, 2, 3, etc.) in order to maintain anonymity of individual health facility results

(4) Describe qualitative information on prescribing obtained from observation and interview with prescribers during the health facility survey, e.g.

- *Qualification of prescriber (doctor, nurse, paramedic);*
- *Number of patients seen per day;*
- *Record keeping (OPD register, prescriptions, recording of diagnosis and drug treatment?);*
- *Prescriber views on quality of prescribing;*
- *Specific examples of irrational / inappropriate prescribing observed in prescriptions.*

Table 6.3.2 show some data on health facility prescription survey.

Table 6.3.2: Results of prescription audit from health facility survey

**Public referral hospitals**	*Insert Name*	*Insert Name*	*Insert Name*	*Insert Name*	Average
Average number of drugs per patient					
% patients prescribed antibiotics					
% patients prescribed injections					
% patients prescribed neutraceuticals*					
% drugs prescribed by generic name					
% prescribed drugs belonging to the EML					
% URTI patients prescribed antibiotics					
Average cost per prescription					
% patients treated with TRM medicines					
**					
**Public district hospitals**	*Insert Name*	*Insert Name*	*Insert Name*	*Insert Name*	Average
Average number of drugs per patient					
% patients prescribed antibiotics					
% patients prescribed injections					
% patients prescribed neutraceuticals*					
% drugs prescribed by generic name					
% prescribed drugs belonging to the EML					
% URTI patients prescribed antibiotics					
Average cost per prescription					
% patients treated with TRM medicines					
**					
**Public primary health care centres**	*Insert Name*	*Insert Name*	*Insert Name*	*Insert Name*	Average
Average number of drugs per patient					
% patients prescribed antibiotics					
% patients prescribed injections					
% patients prescribed neutraceuticals*					
% drugs prescribed by generic name					
% prescribed drugs belonging to the EML					
% URTI patients prescribed antibiotics					
Average cost per prescription					
% patients treated with TRM medicines					
**					

TRM=Traditional Medicine products.
* Neutraceuticals include vitamins, minerals and tonics. *The team should decide whether to collect information on multivitamins and B Complex alone or to include tonics and other minerals.*
** Other medicines use indicator of country's choice e.g. % patients prescribed traditional medicines.

Table 6.3.2 continued: prescription audit from health facility survey

**Private-for-profit pharmacies**	*Insert Name*	*Insert Name*	*Insert Name*	*Insert Name*	Average
Average number of drugs per patient					
% patients prescribed antibiotics					
% patients prescribed injections					
% patients prescribed neutraceuticals*					
% drugs prescribed by generic name					
% prescribed drugs belonging to the EML					
% URTI patients prescribed antibiotics					
Average cost per prescription					
% patients treated with TRM medicines					
**					
**Other facility type***** *Insert type*	*Insert Name*	*Insert Name*	*Insert Name*	*Insert Name*	Average
Average number of drugs per patient					
% patients prescribed antibiotics					
% patients prescribed injections					
% patients prescribed neutraceuticals*					
% drugs prescribed by generic name					
% prescribed drugs belonging to the EML					
% URTI patients prescribed antibiotics					
Average cost per prescription					
% patients treated with TRM medicines					
**					
**Other facility type***** *Insert type*	*Insert Name*	*Insert Name*	*Insert Name*	*Insert Name*	Average
Average number of drugs per patient					
% patients prescribed antibiotics					
% patients prescribed injections					
% patients prescribed neutraceuticals*					
% drugs prescribed by generic name					
% prescribed drugs belonging to the EML					
% URTI patients prescribed antibiotics					
Average cost per prescription					
% patients treated with TRM medicines					
**					

* Neutraceuticals include vitamins, minerals and and tonics. *The team should decide whether to collect information on multivitamins and B Complex alone or to include tonics and other minerals.*
** Other medicines use indicator of country's choice
*** e.g. private hospital, private clinic, public pharmacy, outreach clinic.

6.4. Dispensing Practices

Please describe dispensing practices, from observation and interview, in the pharmacies visited during the health facility survey, remembering the following points:

- *Approximate dispenser-patient interaction time;*
- *Labelling of medicines;*
- *Dispensing records – prescriptions, duplicate prescriptions, bills, registers;*
- *Instructions to patients;*
- *Generic substitution;*
- *Therapeutic substitution (even if against policy and is done due to stock-outs);*
- *Staffing – pharmacists, pharmacy assistants, nurses, unqualified staff;*
- *Dispenser workload (number of patients seen per dispenser per day);*
- *Quality of writing of prescriptions and patient orders;*
- *Storage of medicines;*
- *Observed quality of medicines in the dispensary.*

6.4.1. Health Facility Outpatients

6.4.2. Health Facility Inpatients (wards)

6.4.3. Private retail pharmacies

6.5. Policies to promote rational use of medicines

For each policy in each sub-section, please summarise the information collected from record review, observation and interview with staff at the centre and the periphery.

6.5.1. Monitoring and supervision of prescribing/dispensing by supervisors

Please remember to cover the following points:

- *Is prescription audit, drug utilization review, indicator studies regularly done?*
- *Is there any monitoring of prescribing/dispensing processes, and if so, what is done?*
- *Which government bodies cover monitoring of prescribing and dispensing?*
- *Which staff actually do monitoring (if any is done)?*

6.5.2. Standard Treatment Guidelines (STGs)

Please remember to cover the following points:

- *What different national STGs have been developed?*
- *Do the STGs cover most of the common illnesses at primary care? and secondary care?*
- *Which government bodies have developed STGs?*
- *What distribution has been done and what is the availability of STG in health facilities and on the web?*
- *Are the STGs used in pre-service and in-service training?*
- *Is there consistency between the STGs and national EML?*
- *Are the national STGs used by prescribers? Did any of the prescribers met during the situational analysis have a copy of any national STGs?*
- *Are there STGs covering traditional medicine treatments in primary care?*

6.5.3. National Formulary

Please remember to cover the following points:

- *Is there a national formulary? and if so, which government body developed it?*
- *Does the national formulary include only EML drugs or also non-EML drugs also?*
- *What information provided in the formulary:*
  - *Drug indications, contra-indications, side effects, drug interactions, drug costs, generic names, branded names, drug schedules?*
- *What is the availability of national formulary in health facilities and on the web?*
- *Is the national formulary used in pre-service and in-service training?*
- *Is the national formulary used by prescribers and dispensers? Did any of the health staff met during the situational analysis have a copy of the national formulary?*

6.5.4. Drug information Centre

Please remember to cover the following points:

- *Is there a national drug information centre? If so, which government or non-governmental body runs it?*
- *What services and information are supplied?*
- *What sources of information are used?*
- *What is the frequency of use by end-users according to the centre?*
- *Have any health workers met during the situational analysis used it?*
- *How is the drug information centre funded?*

6.5.5. Independent drug information

Please remember to cover the following points:

- *National clinical guidelines;*
- *Prescriber access to internet;*
- *Visits by medical representatives.*

6.5.6. Drug and Therapeutics Committees (DTCs)

Please remember to cover the following points:

- *Do DTCs exist in most tertiary hospitals? In most secondary hospitals?*
- *What is the usual membership of the DTCs?*
- *What are the usual functions of the DTCs?*
- *What is the usual frequency of DTC meetings?*
- *Do the DTCs get involved in any prescription audit?*
- *Do the hospitals have to report on DTC activities to government or any other quality of care body?*
- *Do the DTCs have a role in antibiotic stewardship programmes?*

6.5.7. Undergraduate education on medicines use

Please remember to cover the following points:

- *Do curricula for medical students and other prescribers include the National EML, National Formulary, National Standard Treatment Guidelines (STGs), problem-based pharmacotherapy, antimicrobial resistance & antibiotic use, and rational prescribing?*
- *Do the curricula for pharmacy students cover clinical pharmacy, good pharmaceutical care and supply management?*
- *What is the role of universities, Ministry of Education and Ministry of Health in setting curricula for different cadres of health staff?*
- *Did any of the referral hospitals visited during the situational analysis have a clinical pharmacy or clinical pharmacology department involved in actual patient care and, if so, how were they involved?*
- *What is the role of health professional bodies and accreditation to ensure quality of care and thus demonstration of best practices to students?*
- *Describe briefly what formal training there is for traditional medicine practitioners.*
- *Are students of conventional medicine taught anything about traditional medicine and are student of traditional medicine taught anything about conventional (allopathic medicine)?*

6.5.8. Continuing Medical Education on medicines use

Please remember to cover the following points:

- *Do curricula include National EML, National Formulary, National Standard Treatment Guidelines (STGs), problem-based pharmacotherapy, and rational prescribing?*
- *How are curricula set and who develops them?*
- *What is the role of universities, Ministry of Education and Ministry of Health in setting curricula for in-service training for different cadres of health staff?*
- *What role do clinical pharmacology and clinical pharmacy play in in-service training?*
- *What is the role of health professional bodies and accreditation in continuing medical education?*
- *Has any CME covered topics on traditional medicine? Antimicrobial resistance and use?*

6.5.9. Public Education on the safe and prudent use of medicines

Please remember to cover the following points:

- *Have any national public education campaigns on prudent use of medicines (including antibiotics) been undertaken in the last 10 years?*
- *What messages on prudent and safe use of medicines (including antibiotics) were spread?*
- *Which government department or other organization was responsible?*
- *What were the channels used (media, community health workers, etc.) and what proportion of the population was covered?*
- *How easy is it for patients to buy prescription-only drugs over-the-counter without prescription (Very easy / easy / possible / impossible)?*

6.5.10. Generic Policies

Please remember to cover the following points:

- *Are there any generic prescribing policies? If so, are they successful?*
- *Is generic substitution legal? Is it undertaken in the public and private sectors?*

6.6. **Summary status including progress / changes / problems** in medicines use since last situational analysis

Have the recommendations from the last situational analysis been acted upon and, if not, why not?

6.7. Medicines use: Recommendations

7. MEDICINE REGULATION

7.1. Responsible Agents/Departments

After discussion with MOH officials, please tick whether the Drug Regulatory Authority (DRA) or another agency is responsible for various functions to regulate medicines and write the name of the agency in the table below.

Regulatory function	DRA	Other Agency	DRA/MOH department/Name of Agency
Drug Schedules			
Licensing & Inspection of manufacturing plants			
Licensing & inspection of wholesale & retail outlets			
Drug licensing or registration			
Pharmacovigilance			
Drug quality testing			
Drug recall			
Clinical trial oversight			
Drug promotion			
Drug pricing			
Health professional licensing/accreditation			
Health facility/hospital licensing/accreditation			

7.2. Pharmaceutical sector

Describe the pharmaceutical sector in box 7.2.1 after discussion with national drug regulatory authority

Box 7.2.1: Summary of the pharmaceutical sector

- Number of products on the market:
  - Allopathic:
  - Traditional:
  - Food supplements with therapeutic claims:
  - Veterinary:
- Number of manufacturers:
  - Allopathic:
  - Traditional:
  - Food supplements with therapeutic claims:
  - Veterinary:
- Number of drug wholesaler outlets:
- Number of retailer pharmacy outlets:
  - Allopathic:
  - Traditional:
- Number of general shops selling:
  - OTC products:
  - Food supplements with therapeutic claims:
- Number of blood outlets *(if under the jurisdiction of the national drug regulatory authority)*:
- Enforcement of regulations in last fiscal year:
  - Number of inspections to manufacturing plants:
  - Number of inspections to wholesale and retail outlets:
  - Number of prosecutions
  - Value of fines
  - Number of people imprisoned

7.3. Current Medicines Legislation[1] (key documentation)

a) Summary of Laws/Regulations in place: *please write the relevant laws in the table*

Name of Law or Regulation	Year

b) Coverage: *indicate with Y (Yes) or N (No) whether the below categories are mentioned in the laws/regulations*

Area / Activity Covered?	Y/N	Document Name
Establishment & functioning of National Drug Regulatory Authority		
Definition of medicines (medicinal products)		
Medicines marketing authorization and licensing		
Medicines scheduling		
Licensing of medicines handling premises, personnel & practices		
Licensing of prescribers		
Mandatory CME for prescriber licence renewal		
Licensing of pharmaceutical personnel		
Mandatory CME for pharmacy licence renewal		
Regulatory inspections/ enforcement activities		
Medicines quality		
Medicines packaging & labelling		
Medicines promotion		
Post-market surveillance/ pharmacovigilance		
Collection of fees		
Clinical trials		
Generic substitution		
TRIPS-related issues		
Transparency & accountability[2]		
Banning of unsafe medicines		

[1] Medicines (medicinal products) regulation issues may be covered in more than one law and may have multiple associated regulations, so ensure that all relevant documentation is identified & obtained for review.
[2] Includes provisions for the Drug Regulatory Authority to define and publish its policies and procedures, publicly account for its decisions, conduct and actions, and follow a regulatory code of conduct.

7.4. National Regulatory Authority for medical products

Box 7.4.1 summarises the functions of the national drug regulatory authority

Please describe the national drug regulatory authority in box 7.4.1 below.

Box 7.4.1. Summary of the National Drug Regulatory Authority

- Name of National Drug Regulatory Authority:
- Total number of technical staff:
  - Number of posts: Number of posts filled:
  - *Please list the different disciplines:*
- Total number of non-technical staff:
  - Number of posts: and number of posts filled:
- Website address:
- Number of quality-control (drug testing) laboratories:
- Annual report of activities? *If yes, give reference:*
- Annual Budget last fiscal year:
- Position in hierarchy of government structure (e.g. under MOH or independent)?
- Decentralised capacity?
  - Number of branch offices:
  - Number of staff in each office:
  - Functions of branch offices: *please list the functions*
- Functions outsourced to public health authorities: *please list the functions*
- Written SOPs for key procedures? *Please answer yes/no and if yes the language of the SOP*
  - Product dossier evaluation?
  - Registration of medicines?
  - Inspection of manufacturing premises?
  - Inspection of retail premises?
  - Sampling for quality control testing?
  - Medical product recall or withdrawal?
  - Oversight of clinical trials
  - Pharmacovigilance

7.4.1. Technical committees to advise the drug regulatory authority

Please describe each committee including:

- *composition of the committee;*
- *roles and responsibilities of the drug regulatory authority versus the committee;*
- *procedures for managing conflict of interest.*

7.4.2. Quality management system

Please describe any quality management system used by the drug regulatory authority

7.4.3. Regulation of Traditional Medicine

Please describe how traditional medicine products and outlets are regulated and whether it is the national drug regulatory authority that is responsible or another agency.

7.5. Drug Schedules

Please remember to cover the following points:

- *Over-the-Counter;*
- *Prescription-only;*
- *Narcotics and controlled drugs;*
- *Schedules according to drug outlet type;*
- *Other categories or schedules;*
- *Traditional medicines;*
- *Documentation;*
- *How easy is it for patients to buy prescription-only drugs over-the-counter without prescription (Very easy / easy / possible / impossible)?*

7.6. Regulation and inspection of drug outlets

7.6.1. Manufacturers

Please remember to cover the following points:

- *Number of GMP inspectors?*
- *Annual Inspection plan?*
- *Number of inspections made in the last one year to manufacturers?*
- *Written Standard Operating Procedures?*
- *Licensing of manufacturers;*
- *Decentralisation of inspections?*

7.6.2. Wholesale and retail outs

Please remember to cover the following points:

- *Number of wholesale and retail outlet inspectors?*
- *Annual inspection plan?*
- *Number of inspections made in the last one year to wholesalers and retailers*
- *Written Standard Operating Procedures?*
- *Licensing of wholesale/retail outlets;*
- *Licensing of professionals operating wholesale and retail drug outlets e.g. pharmacists;*
- *Decentralisation and involvement of public health professions in inspections?*
- *Were the private pharmacies from the health facility survey visited in the last one year?*
- *Inspection of outlets selling traditional medicines.*

7.7. Drug Registration

Please remember to cover the following points:

- *Is there a designated unit? If yes, what is the staffing and budget of the designated unit?*
- *Written Standing Operating Procedures for dossier evaluation and registration process?*
- *Number of products approved in the last 5 years – new active pharmaceutical ingredients (APIs) and APIs already existing in the market;*
- *Description of the process of approval for molecules already on the market with regard to (1) imported drugs (2) locally manufactured drugs;*
- *Description of the process of approval for new molecules;*
- *Number of products de-registered (licence revoked) in the last 5 years – due to expiry of the existing licence or due to safety and/or quality reasons*
- *Membership of the Technical Advisory Committee (that approves registration).*
- *Registration of traditional medicine products.*
- *How many waivers or "No objection letters" to product registration have been issued in the last one year?*

7.8. <u>Pharmacovigilance</u>

Please remember to cover the following points:

- *Is there a designated unit? If yes, what is the staffing and budget of the designated unit?*
- *What problems are covered? ADR monitoring, unexpected lack of efficacy, quality defect, dependence/abuse, medication errors, drug poisoning?*
- *Are other bodies involved in pharmacovigilance? If so, how is the liaison between these bodies and the Drug Regulatory Authority?*
- *Are there written Standard Operating Procedures for recall of products found to be unsafe from pharmacovigilance?*
- *How many problems were reported (ADRs, adverse events) in the last 5 years and what actions were taken? Please fill in table 7.8.1. If information also exists for traditional medicine please get the information and insert into a second table (7.8.2 – which should have the same format as table 7.8.1).*

Table 7.8.1: Number of Adverse Drug Reactions reported at national level in the last 5 years

Year	*Fill the year*	*Fill the year*	*Fill the year*	*Fill the year*	*Fill the year*
Number of ADRs					

7.9. Drug Promotion

Please remember to cover the following points:

- *Is there a designated unit? If yes, what is the staffing and budget of designated unit?*
- *What issues are covered? Pre-approval of adverts, post-approval monitoring of adverts, advert monitoring for OTC drugs, advert monitoring for prescription-only drugs, promotional activities of drug companies including budgets spent on this, access of pharmaceutical representatives to health professionals in public?*
- *How many problems were reported (misleading and unethical adverts) in the last 3 years and what actions were taken?*
- *Are other bodies involved in monitoring drug promotion and, if so, how is the liaison between these bodies and the Drug Regulatory Authority?*
- *Is control of pharmaceutical promotion & advertising in the mass media effective?*

7.10. Drug Price controls

Please remember to cover the following points:

- *Responsible agency?*
- *Type of price control;*
- *Types of drugs with price controls;*
- *Mark-ups by manufacturers, wholesalers, retailers;*
- *Monitoring of prices;*
- *Comment on the price information included in tables 4.3.1 and 4.4.1.*

7.11. Drug Testing Laboratories

Please remember to cover the following points:

- *Name of Drug Testing Laboratory;*
- *Functions of the Drug Testing Laboratory ;*
- *Staffing of the Drug Testing Laboratory;*
- *Laboratory quality standards – measurement standards and quality management standards;*
- *Standing Operating Procedures;*
- *Computerisation of key procedures?*
- *Decentralised capacity?*
- *Capacity to test traditional medicines?*
- *Number of samples of pharmaceutical products tested, from where they were selected and % failing quality standards in the last 5 years and insert data into table 7.11.1. If information also exists for traditional medicine please get the information and insert into a second table (7.11.2 – which should have the same format as table 7.11.1).*

Table 7.11.1: Drug quality testing results for the last 5 years

Year	Samples received		Samples tested		Samples found to be substandard	
	Pre-market authorisation	Post-market authorisation	Pre-market authorisation	Post-market authorisation	Pre-market authorisation	Post-market authorisation
Fill year						
Fill year						
Fill year						
Fill year						
Fill year						

7.12. Drug recall

Describe the drug recall procedures and remember to cover:

- *Number of drug recalls in last 5 years plus reasons (triggers) for the drug recalls;*
- *Remember that reasons for recall may include quality issues arising from laboratory analysis or GMP inspection, safety issues arising from pharmacovigilance, and labelling issues, and that these problems may arise through national or international alerts;*
- *Procedures, including triggers and tracking, for drug recall;*
- *Written Standard Operating Procedures for recall of medicinal (drug) products.*

7.13. Clinical Trial oversight

Please remember to cover the following points for both pre-clinical and clinical trials:

- *Number of clinical trials done in the last 5 years (and whether phase II, III, IV)*
- *Number and licensing of organizations undertaking clinical trials;*
- *Description of the procedures for oversight;*
- *Number of Inspections;*
- *Existence of a national clinical trial registry (database);*
- *Roles and activities of ethics committees.*

7.14. Licensing and accreditation of health professionals

Please remember to cover the following points:

- *Licensing & accreditation of health professionals to practice (doctors, pharmacists, nurses, traditional medicine practitioners);*
- *Licensing & accreditation health facilities/hospitals to operate;*
- *Health professional bodies - Medical Council and Association, Pharmacist Council and Association, Nursing Council and Association, Drug and Chemist Association, Traditional Medicine Council;*
- *Members, Membership fees, activities of health professional bodies.*

7.15. Licensing and accreditation of health facilities and pharmacies

Please remember to cover the following points:

- *Licensing and accreditation health facilities/hospitals/pharmacies/traditional medicine clinics to operate;*
- *Body regulating health facilities/hospitals/traditional medicine clinics and their quality of care.*

7.16. **Summary status including progress / changes / problems** in medicines regulation since last situational analysis

Have the recommendations from the last situational analysis been acted upon and, if not, why not?

7.17. Medicines regulation: Recommendations

8. MEDICINE POLICY AND COORDINATION

8.1. National Medicines Policy

Please remember to cover the following points:

- *Document reference;*
- *Aims of the national medicines policy;*
- *What aspects of medicines management are covered in the policy?*
- *Is there a policy implementation plan and budget?*

8.2. Summary of medicines policies in place to promote rational use of medicines

After discussion with MOH officials, and based on your observations, please indicate whether the various policies mentioned in the table below are implemented or not.

Policy	Implementation status
National Medicines Policy (NMP)	
National Essential Medicines List (EML)	
National Standard Treatment Guidelines (STGs)	
National Formulary manual	
National government unit dedicated to promoting rational use of medicines	
Monitoring medicines use	
Drug and Therapeutic Committees (DTCs)	
National Drug Information Centre (DIC)	
Generic Policies	
Health insurance	
Payment for medicines by patients	
Provider revenue from medicines	
Undergraduate training on pharmacology & prescribing	
CME training on pharmacology & prescribing	
Public education on medicines use	
Pharmacovigilance	
Regulation of drug promotion	
National strategy to contain Antimicrobial Resistance	
Over-the-counter availability of prescription-only medicines including antibiotics	

8.3. Coordination of medicines-related policies within the Ministry of Health

8.3.1. Ministry of Health Organogram

Please draw a Ministry of Health Organogram

8.3.2. Coordination within the Ministry of Health

Please remember to cover the following points:

- *Which departments have functions related to medicines and traditional medicine?*
- *What functions do the different departments cover?*
- *How is coordination managed?*

8.4. Other Ministries with medicines-related functions

Please remember to cover the following points:

- *List all the different Ministries plus their functions e.g. Ministry of Finance (budget); Ministry of Trade & Industry (pharmaceutical manufacturers); Ministry of Education (health professional training); Ministry of Commerce (drug prices); Public Services Commission (human resources)*
- *What functions do the different Ministries cover?*
- *How is coordination managed?*

8.5. **Summary status including progress / changes / problems** in medicines policy since last situational analysis

Have the recommendations from the last situational analysis been acted upon and, if not, why not?

8.6. Medicines policy and coordination: Recommendations

9. References

Please list all references cited in the text of the report.

10. PERSONS MET DURING THE SITUATIONAL ANALYSIS

Please fill the names of all persons met during the situational analysis with designation, affiliation and contact details. Once the report is finalized, contact details should be deleted prior to publication. Please add pages as needed.

	Name	Designation and Affiliation	Contact details
1			
2			
3			
4			
5			
6			
7			
8			
9			
10			
11			
12			
13			
14			
15			
16			
17			
18			
19			
20			
21			
22			
23			
24			
25			
26			
27			
28			
29			
30			

11. PARTICIPANTS OF THE STAKEHOLDER WORKSHOP

Please fill the names of all persons participating in the stakeholder workshop with designation, affiliation and contact details. Once the report is finalized, contact details should be deleted prior to publication. Please add pages as needed.

	Name	Designation and Affiliation	Contact details
1			
2			
3			
4			
5			
6			
7			
8			
9			
10			
11			
12			
13			
14			
15			
16			
17			
18			
19			
20			
21			
22			
23			
24			
25			
26			
27			
28			
29			
30			

12. WORKSHOP SLIDE PRESENTATION

Please import the slide presentation made at the national workshop in double columns below.

13. HEALTH FACILITY SURVEY FORMS

Facility Name:

District/Province:

Level of Health Care:

Date:

Please photocopy extra sets of forms for use - one set of forms for each health facility visited

13.1. Introduction

Please meet the person in charge of the health facility to explain your purpose in visiting the facility. Choose one person from the team to explain that you wish to learn about how medicines are managed in the health facility and that you are not here to make any judgments and that what staff members tell you will be treated in confidence.

Please explain that you will be holding a national stakeholder workshop and that 1-2 persons from that facility are invited to participate. At the workshop the findings overall (not for individual facilities) from the situational analysis will be discussed & recommendations made for future action.

Please then fill in section 13.2.

13.2. Health Facility Identification

Health Facility name: _______________ Province/Region/District: ________________ Date: __________

Health facility type: referral hospital /district hospital /primary care centre /sub-centre *Please circle*

Health facility ownership: Public / private-for-profit / private not-for-profit *Please circle*

13.2.1. Respondents interviewed

Please complete the table with all interviewee names after finishing the survey

Name	Designation

*Then explain that you would like to divide into **3 groups**:*

- ***Group 1**: to interview the health facility in-charge and the administrative staff to: ask about administrative and organizational details (section 13.3); to observe patient flow in the out-patients (section 13.4.1) including review of any outpatient register (with patient diagnosis and treatment, section 13.4.2) and to review ward & inpatient drug management. (section 13.4.3).*
- ***Group 2:** to visit the main pharmacy and drug store to review drug availability (section 13.5);*
- ***Group 3:** to visit the outpatient pharmacy to observe dispensing practices and undertake a prescription survey of general outpatient cases (section 13.6).*

13.3 **Administration** – from health facility in-charge and administrator (Group1)

Health Facility name: _______________ Province/Region/District: ________________ Date: __________

Interviewer (team) names: ____

Interviewee (facility staff) names: ____

13.3.1. Catchment area and utilization levels

Population served: __________________

Number of beds: ______ Number of inpatients today: ________________ Bed occupancy:________

OPD visits per day (average): new _________ old cases __________ total _________________

OPD visits per month (average): new _______ old cases __________ total _________________

Traditional medicine: OPD visits per month (average): new ______ old cases_______ total_________

13.3.3. Staffing

Type of Staff	Number of Posts	Number of staff in post	Number of staff present today
Specialist doctors			
General doctors			
Nurses			
Pharmacists			
Pharmacy assistants			
Store-keepers for medicines			
Administrators			
Paramedics (specify)			
Traditional medicine practitioner			
Other (specify) involved in medicine management			

Is staffing sufficient? Yes / No *Please circle*

If no, what extra staff are needed?

13.3.4. Funding of medicines

Source of funds	Yes/No	% of total budget
Central MOH budget allocation		
Local MOH budget allocation		
Cost recovery fees income		
Other (specify):		

Are funds for medicines sufficient? Yes / No *Please circle*

If no, explain how you cope with this:

13.3.5. Fees that patients must pay

Services charged for	Charged Yes/No	Describe Type of fee	How are the fees used?
Registration fee			
Outpatient consultation fee			
Inpatient bed fee			
Fees for outpatient drugs			
Fees for inpatient drugs			
Laboratory fee			
Surgical dressing fee			
Other fees (describe)			

13.3.6. Health Insurance

What proportion of your patients has health insurance? None / Few / Half / Most / All *Please circle*

Cost item	Covered **P**artially or **F**ully?	If partial, describe co-payments
Consultation		
Laboratory Services		
Out-patient Medicines		
In-patient Services		
In-patient Medicines		
Other (specify)		

Does your facility get payment for medicines dispensed to insured patients from the patients (who then claim back from the insurance company) or from the insurance company?

13.3.7. Drug and Therapeutics Committee

Is there a hospital DTC? Yes/No *Please circle*

If yes, please describe the DTC, remembering to cover the following points:

- *Chairman, Secretary, and membership*
- *Activities in the last one year*
- *Budget for activities*
- *Frequency of meetings, date of last meeting*
- *Use of Formulary manual and clinical guidelines*
- *Drug policies (e.g. automatic stop orders, structured order forms, 3-day prescriptions, procurement)*
- *Monitoring medicines use (e.g. prescription audit and feedback, drug utilization review)*
- *Monitoring Adverse Drug Reactions and Adverse Drug Events*
- *In-service training*

13.3.8. Regulation/inspection

Has an inspection of the facility been done in the last one year? Yes/No *Please circle*

If yes, please describe the regulation/inspection, remembering to cover the following points:

- *When did you last receive an inspection?*
- *Who carried out the inspection?*
- *What was inspected? Pharmacy? Hospital services?*
- *What feedback was given?*

13.3.9. Problems in drug management

Ask the person in charge of the health facility (e.g. Hospital Director, Health Post-in-Charge) and the administrator what problems they face in drug management.

13.3.10. Problem in prescribing

Ask the person in charge of the health facility (e.g. Hospital Director, Health Post-in-Charge) and the administrator what problems they face in drug prescribing by doctors and health workers.

13.4 **Patient Flow** from visiting outpatient department and some wards (Group 1)

Health Facility name: ________________ Province/Region/District: ______________ Date: _________

Interviewer (team) names: ____

Interviewee (facility staff) names: ____

13.4.1. General (non-specialist) Outpatients

Visit the OPD and answer the below questions

- What cadres of staff are prescribing in outpatients today? ____________________________________
- How is triaging (screening) of patients done? ____
- Is there crowding of patients (i.e. more than one patient plus family around one prescriber)? Yes/No
- What documents are available in the Outpatient Department? STGs Yes/No ; EML Yes/No *Circle*
  - What STGs are available (if any)? ____
- Describe the OPD register: Is diagnosis recorded? Yes/No Drug Treatment? Yes/No *Circle*
  - Who fills it in? Prescriber (e.g. doctor) or an assistant (e.g. nurse): ________________________
- Describe the prescriptions: Is diagnosis recorded? Yes/No *Circle*
  - Are separate prescriptions written for drugs to be purchased in outside pharmacies? Yes/No
  - What is the quality of prescription writing? ____

Interview briefly a few prescribers in the OPD and fill in the table below.

Cadre of prescriber in OPD*	No. patients/ day seen	Dates of CME in last year	Subjects covered in CME	Used DIC in last year? Y/N	Seen Med Rep in last month? Y/N
1.					
2.					
3.					

* doctor, nurse, paramedical worker, other. DIC = Drug Information Centre

Review the documentation in the consultation rooms of a few prescribers, discuss with them, and fill in the table below.

Cadre of prescriber in OPD*	OPD register (drugs recorded in >80% cases)	OPD register (diagnosis recorded in >80% cases)	STG /EML available in consulting room?	Other documentation? e.g. separate drug slips	No. patients/day given prescriptions to buy drugs in outside pharmacies
1.					
2.					
3.					

* doctor, nurse, paramedical worker, other. STG = Standard Treatment Guidelines

13.4.2. Inpatients *Visit 2-3 general wards and answer the questions below.*

Ward name	Ward type	No. beds	No. in-patients today	No. nurses in the ward now	No. patients/day buying drugs from outside pharmacies	Sink with running water and soap present in the ward	Clean Yes/No?

Ward name	Ward type	List drugs out of stock

- Describe the inpatient records: e.g. forms, numbered tags, dispensing records:

- Describe the dispensing process to inpatients:

- Describe the ward drug stores

13.5. <u>Drug Management</u> – from pharmacy in health facility (Group2)

Health Facility name: ________________ Province/Region/District: ______________ Date: _________

Interviewer (team) names: ____

Interviewee (facility staff) names: ____

13.5.1. Drug Procurement

Please circle

- Where are drug supplied from? Centre Yes/No District/province Yes/No Local purchase? Yes/No
- If there is local procurement,
  - Approximately what proportion of the total drug budget is on local purchase?
  - What is the process of drug procurement? *Remember to include the procurement rules.*

13.5.2. Drug Selection

Please circle

- Where is the decision taken about which drugs are on the list? Centre Yes/No Locally Yes/No
- If you decide locally, what is the process?

13.5.3. Drug Quantification

- How do you estimate the quantity of medicines needed?

  Remember to include details of any formula use and what buffer stock is maintained if any

13.5.4. Ordering and re-distribution to other health facilities

Function	Routine ordering and redistribution to the health facilities *Please tick relevant box*					Number of emergency orders in last one month
	weekly	monthly	3-monthly	6-monthly	annual	
Ordering from suppliers						
Re-distribution to health facilities						

13.5.5. Describe the store conditions

13.5.6. What are your sources of drug information?

Please circle

- Medical representative visits? Yes/No If yes, when was the last visit? ______________
- National drug information centre? Yes/No If yes, when did you last use them?___________
- Access to the internet? Yes/No If yes, when did you last use it?______________
- MIMS, National Formulary, EML, other books (specify)____

13.5.7. Problems in drug management

Ask the pharmacist or staff member in charge of the pharmacy what problems she or he faces in drug management.

13.5.8. Stock availability of essential items:

Facility name:___________________

Fill in the drug names that have been selected from the EML and mark whether they are available or not. Even if one bottle or vial or a few tablets are present, the drug should be marked available.

#	Drug Name	Formulation	Strength	Available? Y/N	Unit Price
1					
2					
3					
4					
5					
6					
7					
8					
9					
10					
11					
12					
13					
14					
15					
16					
17					
18					
19					
20					
21					
22					
23					
24					
25					
26					
27					
28					
29					
30					
31					
32					
33					
34					
35					
36					
37					
38					
39					
40					
% availability of (1) all items, (2) items that should be available in facility					

13.5.9. Stock-outs:

Facility name:____________________

List all the drugs items out of stock today and, if possible, the number of days out of stock (O/S). Please also list the total number of items on the procurement list.

#	Drug Name	Formulation	Strength	Number of days O/S
1				
2				
3				
4				
5				
6				
7				
8				
9				
10				
11				
12				
13				
14				
15				
16				
17				
18				
19				
20				
21				
22				
23				
24				
25				
26				
27				
28				
29				
30				

Total number of drug items on the procurement or order list for the facility? _________

13.5.10. Expired items:

Facility name: ___________________

List all the drug items that have expired in the last fiscal year and, if possible, the date they expired and their monetary value.

#	Drug Name	Formulation	Strength	Date expired	Monetary value
1					
2					
3					
4					
5					
6					
7					
8					
9					
10					
11					
12					
13					
14					
15					
16					
17					
18					
19					
20					
21					
22					
23					
24					
25					
26					
27					
28					
29					
30					

13.5.11. Stock utilization: ABC analysis of top 20 items: Facility name: _____________

List all the drug items in descending order of monetary value for the last fiscal year.

Source of data (procurement/distribution): _________________________ Year ___________

Rank	Drug Name	Formulation	Strength	EML Y/N	Unit cost	Units used	Monetary Value
1							
2							
3							
4							
5							
6							
7							
8							
9							
10							
11							
12							
13							
14							
15							
16							
17							
18							
19							
20							

- Total number of items on hospital/facility procurement/order list:
- Total value of all items procured/distributed (not just top 20):
- % of budget consumed by top 20 drugs by value:
- % of total budget value due to:
    - Antibiotics:
    - Vitamins/minerals/neutraceuticals *Please circle*:
    - National EML drugs:

13.6. **Dispensing** – from observation in the outpatient pharmacy (Group 3)

Health Facility name: _______________ Province/Region/District: ___________________ Date: __________

Interviewer (team) names: ____

Interviewee (facility staff) names: ____

13.6.1. Dispenser type: *Please circle*

Pharmacist / qualified pharmacy assistant / unqualified pharmacy assistant / nurse / paramedic / other

13.6.2. Dispensing records and equipment: *Please circle*

- Dispensing book or register? Yes / No   Other documentation? ____________________
- Are tablets counted by hand? Yes / No   Is a tablet counter used? Yes / No
- What containers are used for dispensed tablets / capsules? _______________________________

13.6.3. Labelling:

Type of label used for dispensed items (tick each box as applicable)

Item	Hand-written	Printed	Self-adhesive	Other adhesive	No label
Tablets/capsules					
Oral liquids					
Ointments & creams					
Injections					
Other (specify)					

- What information is give on the label? *Please circle*

  Patient's name: Yes/No   Drug generic name: Yes/No   Drug strength: Yes/No

  Dose size: Yes/No   Dose frequency: Yes/No   Treatment duration: Yes/No

  Other information: ____

13.6.4. Dispenser-patient interaction: *Please circle*

- Observe the dispenser-patient interaction time in 10 patients to estimate average duration:

  < 1 minute / 1-2 minutes / 3-4 minutes / > 5minutes   *Please circle*

13.7. <u>Prescribing</u> – from observation in the outpatient department or outpatient pharmacy and review of prescriptions (Group 3)

Health Facility name: _______________ Province/Region/District: ________________ Date: __________

Interviewer (team) names: ____

Interviewee (facility staff) names: ____

The team must identify where data may be collected very early in the health facility visit. After the initial introduction to the health facility in-charge, the team leader should go with the team to the hospital outpatient department (OPD) and pharmacy to determine the sources of data, what data is available, and where data collection should be done.

If all medicines (whether or not dispensed from the health facility) are written on the same prescription slip, and the pharmacy keeps a copy of the prescriptions with an indication of what medicines have been dispensed, then data may be collected retrospectively from the latest prescriptions stored in the OPD pharmacy. However, if separate prescriptions are written for medicines to be dispensed from the health facility as opposed to medicines to be purchased from outside pharmacies and/or if the pharmacy OPD does not keep a copy of the patient prescription, then data should be collected prospectively by asking patients to see the prescription(s) they have been given. The place where patients are stopped for review of their prescriptions could be at the OPD pharmacy or in the OPD depending on logistical convenience.

Only the prescriptions of primary care type cases or general cases (medical and paediatrics), not the prescriptions of specialist cases, should be reviewed. This means that in referral hospitals the team must ask the health facility staff how best to manage this. It could be that the OPD pharmacy staff identify which prescriptions come from specialist versus general clinics or it may be that the data collectors must place themselves in the areas where patients come out of general clinics.

Every effort should be made to visit health facilities during the opening times of outpatient departments, as otherwise a prescribing survey may not be possible. In some primary health care facilities with well-maintained OPD registers and full dispensing of medicines from the facility, data may be collected retrospectively from the OPD register rather than prescriptions, after the closing time for patient consultation. However, in hospitals OPD registers are often poorly maintained, so often data can only be collected prospectively through review of patient prescriptions.

Two sets of forms (31.7.2) are provided to allow to data collectors to work simultaneously.

13.7.1. Prescriber type:

Doctor/ nurse / paramedic / unqualified / Other (specify)______________________________

Please circle

13.7.2. OPD Prescription Data Collection Form (general/PHC patients only) Dates of prescriptions: ________ Health facility name: ______________

Source of data: prescriptions / outpatient register / patient records *Please circle*

#	Diagnosis	Age (yrs)	No. items	No. generics	TRM (Y/N)	AB (Y/N)	INJ (Y/N)	VIT (Y/N)	No. items on EML	No. items dispensed	Drug names	Cost per prescription
1												
2												
3												
4												
5												
6												
7												
8												
9												
10												
11												
12												
13												
14												
15												
16												

#	Diagnosis	Age* (yrs)	No. items	No. generics	TRM (Y/N)	AB (Y/N)	INJ (Y/N)	VIT (Y/N)	No. items on EML	No. items dispensed	Drug names (preferably generic names but brand names if generic names unknown)	Cost per prescription
17												
18												
19												
20												
21												
22												
23												
24												
25												
26												
27												
28												
29												
30												
Totals												
Average												
%				% of total items	% of total items	% of total cases	% of total cases	% of total cases	% of total items	% of total items		

*If <1 year indicate as follows: 3 months = 3/12, 5 months = 5/12, etc.; TRM=Traditional Medicine; AB=Antibiotic; INJ=Injection; VIT=Vitamin

13.7.2. OPD Prescription Data Collection Form (general/PHC patients only)   Dates of prescriptions: ________ Health facility name: _____________

Source of data: prescriptions / outpatient register / patient records *Please circle*

#	Diagnosis	Age (yrs)	No. items	No. generics	TRM (Y/N)	AB (Y/N)	Inj (Y/N)	Vit (Y/N)	No. items on EML	No. items dispensed	Drug names	Cost per prescription
1												
2												
3												
4												
5												
6												
7												
8												
9												
10												
11												
12												
13												
14												
15												
16												

#	Diagnosis	Age* (yrs)	No. items	No. generics	TRM (Y/N)	AB (Y/N)	Inj (Y/N)	Vit (Y/N)	No. items on EML	No. items dispensed	Drug names (preferably generic names but brand names if generic names unknown)	Cost per prescription
17												
18												
19												
20												
21												
22												
23												
24												
25												
26												
27												
28												
29												
30												
Totals												
Average												
%				% of total items	% of total items	% of total cases	% of total cases	% of total cases	% of total items	% of total items		

*If <1 year indicate as follows: 3 months = 3/12, 5 months = 5/12, etc.; TRM=Traditional Medicine; AB=Antibiotic; INJ=Injection; VIT=Vitamin; Px=Prescription

13.7.3. Examples of inappropriate prescribing: Facility Name____________

While doing the prescription survey (section 13.7.2), please note down examples of poor prescription writing, inappropriate indications, drug interactions, incorrect dosing.

	Prescriber type*	Example of inappropriate prescription
1		
2		
3		
4		
5		
6		
7		
8		
9		
10		

* Senior doctor, junior doctor, nurse, paramedic, pharmacist, pharmacy assistant, unqualified person

13.7.4. Antibiotic use in acute upper respiratory tract infection Facility Name: ___________

Source of data: prescriptions / outpatient register / patient records *Please circle*

Identify 30 upper respiratory tract infection (URTI) cases from prescriptions or from the OPD register depending on where diagnosis is recorded. For each patient, note the URTI type, any other diagnosis, record all drugs and mark whether antibiotics (AB) were given or not (Yes/No). URTI =cough, cold, rhinitis, sore throat, pharyngitis, acute otitis media, non-pneumonia, acute bronchitis.

Patient	URTI type	Other diagnosis	All drugs prescribed (incl. antibiotics)	AB (Y/N)
1				
2				
3				
4				
5				
6				
7				
8				
9				
10				
11				
12				
13				
14				
15				
16				
17				
18				
19				
20				
21				
22				
23				
24				
25				
26				
27				
28				
29				
30				
% total				

14. PUBLIC HEALTH OFFICE OR DRUG WAREHOUSE SURVEY FORMS

Public Health Office/Warehouse Name:

District/Province:

Date:

Please photocopy extra sets of forms for use - one set of forms for each warehouse visited

14.1. Public Health Office / Warehouse Identification

Public health office/warehouse name: ____ Date: ___________

Province/Region/District: _____________________ Population served: _____________________________

Number of health facilities supplied: ____

Interviewer (team) names: ____

14.1.1. Respondents interviewed

Name	Designation

14.1.2. Staffing

Type of Staff	No. Posts	No. staff in post	No. staff present today
Doctors			
Public Health specialists			
Pharmacists			
Pharmacy assistants			
Store-keepers			
Administrator			
Other (specify)			

Is staffing sufficient? Yes / No *Please circle*

If no, what extra staff are needed?

14.2. Drug Management

14.5.4. Funding of medicines

Source of funds	Value in last fiscal year	% of total budget
Central MoH budget allocation		
Local MoH budget allocation		
Cost recovery fees income		
Other (specify):		

Are funds for medicines sufficient? Yes / No *Please circle*

If no, explain how you cope with this:

14.5.5. Drug Procurement

- Where are drug supplied from? Centre Yes/No Local purchase? Yes/No *Please circle*
- If there is local procurement:
  - Approximately what proportion of the total drug budget is on local purchase?
  - What is the process of drug procurement? *Remember to include the procurement rules.*

14.5.6. Selection of drugs for the procurement list

Please circle

- Where is the decision taken about which drugs are on the list? Centre Yes/No; Locally Yes/No
- If you decide locally, what is the process?

14.5.7. Drug Quantification

How do you estimate the quantity of medicines needed? *Does it include buffer stock are formulae used?*

14.5.8. Ordering and distribution schedules

Function	Routine ordering and distribution *Please tick relevant box*					Number of emergency orders in last one month
	weekly	monthly	3-monthly	6-monthly	annual	
Ordering from suppliers						
Distribution to health facilities						

14.5.9. Describe the store conditions

14.5.10. Stock availability of essential items: Warehouse name_______________

Fill in the drug names that have been selected from the EML & mark whether they are available or not. Even if one bottle / vial or a few tablets are present, the drug should be marked available.

#	Drug Name	Formulation	Strength	Available? Y/N	Unit Price
1					
2					
3					
4					
5					
6					
7					
8					
9					
10					
11					
12					
13					
14					
15					
16					
17					
18					
19					
20					
21					
22					
23					
24					
25					
26					
27					
28					
29					
30					
31					
32					
33					
34					
35					
36					
37					
38					
39					
40					
	% availability of all items				

14.5.11. Stock-outs at warehouse: Warehouse name: _______________

List all the drugs items out of stock today and, if possible, the number of days out of stock (O/S). Please also list the total number of items on the procurement list.

#	Drug Name	Formulation	Strength	Number of days O/S
1				
2				
3				
4				
5				
6				
7				
8				
9				
10				
11				
12				
13				
14				
15				
16				
17				
18				
19				
20				
21				
22				
23				
24				
25				
26				
27				
28				
29				
30				

Total number of drug items on the procurement or order list? _________

14.5.12. **Expired items at warehouse:** Warehouse name: _______________

List all the drug items that have expired in the last fiscal year and, if possible, the date they expired and their monetary value.

#	Drug Name	Formulation	Strength	Date expired	Monetary value
1					
2					
3					
4					
5					
6					
7					
8					
9					
10					
11					
12					
13					
14					
15					
16					
17					
18					
19					
20					
21					
22					
23					
24					
25					
26					
27					
28					
29					
30					

14.5.13. Stock utilization: ABC analysis of top 20 items

Warehouse name: ________

List all the drug items in descending order of monetary value for the last fiscal year.

Source of data (procurement/distribution): ________________________________ Year ____________

Rank	Drug Name	Formulation	Strength	EML Y/N	Unit cost	Units used	Monetary Value
1							
2							
3							
4							
5							
6							
7							
8							
9							
10							
11							
12							
13							
14							
15							
16							
17							
18							
19							
20							

- Total number of items on hospital/facility procurement/order list: ______
- Total value of all items procured/distributed (not just top 20): __________
- % of budget consumed by top 20 drugs by value: _______
- % of total budget value due to: Antibiotics _____ Vitamins/neutraceuticals _____ EML drugs ______
- Per capita expenditure on medicines in last fiscal year: _______

15. RETAIL PHARMACY SURVEY FORMS

Pharmacy Name:

District/Province:

Date:

Please photocopy extra sets of forms for use - one set of forms for each retail pharmacy visited

15.1. Retail Pharmacy Identification

Retail Pharmacy name: _______________ Province/Region/District: ________________ Date: __________

Interviewer (team) names: ____

15.1.1. Respondents interviewed

Name	Designation

15.1.2. Staffing

Type of Staff	No. persons employed	No. persons present today
Retail Pharmacy Owner		
Doctors in on-site attached clinic		
Pharmacist		
Trained pharmacy assistant		
Untrained pharmacy assistant		
Paramedics (specify)		
Other (specify)		

- Is the pharmacy owner a qualified pharmacist or pharmacy assistant? Yes/No *Please circle*

15.1.3. Organisation

- Opening hours: Approximate daily sales (in local currency):
- Number of nearby pharmacies: Approximate number of patients per day:
- What type of client does this retail pharmacy mainly serve?
  - Hospital patients / Patients from private doctors / Self-medicating patients *Please circle*

15.2. Drug Management

- Approximately, how many products do you have on your shelves?
- How many suppliers do you generally buy from?
- How frequently do you order medicines? Daily/every few days/weekly/monthly *Please circle*
- How frequently do company representatives visit? Daily/every few days/weekly/monthly
  - Do they bring stock with them? Yes/no *Please circle*
  - Do they provide samples of new drugs? Yes/No *Please circle*
- Do you have an electronic drug management information system? Yes/No *Please circle*
- Are most (more than 80% of clients) paper patient bill receipts kept? Yes/ No *Please circle*
- How are drugs stored? Alphabetically/ Therapeutic class/ Formulation type/ Other *Please circle*
  - If other, please describe *(including no system if applicable)*:
- Describe the storage conditions?
- List any expired items found on the shelves

15.2.1. Stock availability of essential items

Retail pharmacy name: ________________

Fill in the drug names that have been selected from the EML & mark whether they are available or not. Even if one bottle / vial or a few tablets are present, the drug should be marked available.

#	Drug Name	Formulation	Strength	Available? Y/N	Unit Price
1					
2					
3					
4					
5					
6					
7					
8					
9					
10					
11					
12					
13					
14					
15					
16					
17					
18					
19					
20					
21					
22					
23					
24					
25					
26					
27					
28					
29					
30					
31					
32					
33					
34					
35					
36					
37					
38					
39					
40					
	% availability of all items				

15.2.3. Out-of-stock items

Please ask the retailer what items are out of stock and record them

15.2.4. Expired items

Please list any expired items found on the shelves

15.2.5. Stock Utilisation

Please ask the retailer what his top 20 drugs by sales (monetary value) are in the last one month. If the retailer has an electronic management information system, he may be able to give this information from his/her computer. Otherwise, just note his/her opinion about what he/she feels are the top 20 drugs by sales (monetary value).

15.3. <u>**Dispensing**</u> – from observation in the retail pharmacy

Health Facility name: _______________ Province/Region/District: ________________ Date: __________

Interviewer (team) names: ____

15.3.1. Dispenser type: *Please circle*

Pharmacist / qualified pharmacy assistant / unqualified pharmacy assistant / nurse / paramedic

15.3.2. Dispensing records and equipment: *Please circle*

- Dispensing book or register? Yes / No Other documentation? _____________________
- Are tablets counted by hand? Yes / No Is a tablet counter used? Yes / No
- What containers are used for dispensed tablets / capsules? ________________________________

15.3.3. Labelling:

Type of label used for dispensed items (tick each box as applicable)

Item	Hand-written	Printed	Self-adhesive	Other adhesive	No label
Tablets/capsules					
Oral liquids					
Ointments & creams					
Injections					
Other (specify)					

- What information is give on the label? *Please circle*

  Patient's name: Yes/No Drug generic name: Yes/No Drug strength: Yes/No

  Dose size: Yes/No Dose frequency: Yes/No Treatment duration: Yes/No

  Other information: ____

15.3.4. Dispenser-patient interaction: *Please circle*

- Observe the dispenser-patient interaction time in 10 patients to estimate average duration:

  < 1 minute / 1-2 minutes / 3-4 minutes / > 5minutes *Please circle*

15.3.5. Pharmacy Client Data Collection Form Retail Pharmacy name: ______________________________ Date: _____________

Data collected from: prescriptions / dispensing register / computer registry / bills *Please circle*

#	Diagnosis	Age (yrs)	Px Y/N	No. items	No. generics	No. TRM	AB (Y/N)	INJ (Y/N)	VIT (Y/N)	No. items on EML	No. items dispensed	Drug names (preferably generic names but brand names if generic names unknown)	Cost per Px
1													
2													
3													
4													
5													
6													
7													
8													
9													
10													
11													
12													
13													
14													
15													
16													

#	Diagnosis	Age* (yrs)	Px Y/N	No. items	No. generics	No. TRM	A/b (1/0)	Inj (1/0)	Vit (1/0)	No. items on EML	No. items dispensed	Drug names (preferably generic names but brand names if generic names unknown)	Cost per Px
17													
18													
19													
20													
21													
22													
23													
24													
25													
26													
27													
28													
29													
30													
	Totals												
	Average												
	%		% of total cases		% of total items	% of total items	% of total cases	% of total cases	% of total cases	% of total items	% of total items		

*If <1 year indicate as follows: 3 months = 3/12, 5 months = 5/12, etc.; TRM=Traditional Medicine; AB=Antibiotic; INJ=Injection; VIT=Vitamin; Px=Prescription

16. PREPARATION

16.1. Preliminary consultations

- With MOH officials and other stakeholders;
- Explain that the situational analysis is an exercise to learn about the management of medicines in health care delivery and to identify priority problems and possible solutions.
- Agree the exact terms of reference;
    - to undertake a rapid situational analysis of medicines in health care delivery, including a national stakeholder workshop;
    - with or without any particular focus or other component as agreed with the MOH.
- Two-week process involving visits to:
    - all the major government departments responsible for drug supply, selection, use, regulation and policy;
    - at least one major public university to discuss education in medicine, pharmacology and pharmacy;
    - at least 2 provinces and in each province to visit 1-2 public referral hospital, 2 public district hospital, 2 public primary health care facilities and 2-4 private-for-profit pharmacies (i.e. minimum of 14-20 facilities); in addition visits to 1-2 private clinics/hospitals may be done.
    - health professional bodies (council and organization);
    - non-governmental organizations involved in management of medicines.

16.2. Distribution of this workbook tool

- The workbook tool should be distributed in hard and soft copy to all government team members in advance. Also one copy of each facility survey form (sections 13-15) for each facility to be visited should be photocopied ready for use during data collection.

16.3. Authorization and approvals

- In advance from MOH and other Ministries if necessary and for all facilities to be visited.

16.4. Identification of key stakeholders and respondents

- Ministry of Health and other relevant Ministries according to the country;
- Departments responsible for drug supply (procurement and distribution), drug selection, drug use, drug regulation and drug policy.

16.5. Budget

- Must be agreed in advance to cover all activities as listed below, including:
    - Transport and accommodation of the assessment team, incl. international & national consultants;
    - Stakeholder workshop at the end of the situational analysis;
    - Transport and accommodation of government staff to attend the workshop;
    - Publication of the report.

16.6. Assembly of assessment team

- At least 5 government officials, one expert in each of drug supply, drug selection, drug use, drug regulation and drug policy and to include middle level pharmacists and doctors;
- The team members should participate throughout the 2-week process, including the meeting to prepare for the workshop, and if absent for some days should be replaced by a another staff member from the same department;
- Team members should ideally do some homework by filling in parts of the tool in advance of the situational analysis.

16.7. Arrangement for coordination and supervision

- At least one senior international consultant is needed to provide technical input and facilitate discussion between different bodies responsible for different aspects of medicines management.
- One national consultant which may be the focal person in the WCO is needed to make administrative arrangements in advance.
- The national consultant should distribute the tool to all government team members in advance.

16.8. Identification and location of key literature

- National Medicines Policy document;
- National Essential Medicines List;
- National Standard Treatment Guidelines;
- Legislation on drug regulation;
- Reports on drug availability, drug use;
- MOH annual reports on utilization, drug supply, drug use, morbidity;
- Drug Regulatory Annual reports.

16.9. Arrangements for health facility surveys

- Written Permissions for each and every facility to be visited;
- Schedule of visits with adequate travel time between facilities;
- Explain that in each facility the team will need to:
    - see all records pertaining to medicines management including stock records, dispensing records, at least 30 prescriptions, and doctors' patient registers;
    - talk with all people involved in the management of medicines, including doctors, pharmacists and other staff involved in management of medicines.
- Each facility requires a minimum time for a visit as follows:
    - Referral hospital – 4 hours;
    - District hospital – 3 hours;
    - Primary health centre (2 hours);
    - Private pharmacy (2 hours if there is an electronic bill system or bill copies but 4 hours if reliant on waiting for patients to see how dispensing is undertaken).

16.10. 1-day Stakeholder workshop

- Takes place at the end of the situational analysis
- Participants should include senior MOH officials and other stakeholders including NGOs, donors, other partners, representing all aspects of drug management (drug supply, selection, use, regulation, policy) and including representatives from all the provinces visited
- Consists of:
  - presentation of findings, progress since last situational analysis, identification of priority problems and suggested solutions
  - plenary discussion for clarification and validation of findings
  - group work to discuss solutions and form recommendations
  - plenary discussion to finalize recommendations and plans of action for their implementation.

16.11. Situational Analysis Report

- Should be written by the assessment team and submitted to MOH within one month
- Based on this workbook and including recommendations from the workshop
- Should be finally approved by MOH.

16.12. Confidentiality

- Maintaining the confidentiality of who said what is very important to avoid later repercussions against people who may have said unpopular things.
- No view or prescribing detail should be ascribed to any specific prescriber either in the workbook or during presentations or discussions in the stakeholder workshop. All facility names should be deleted from the report before finalization.
- Confidentiality in the workbook is maintained by recording the names, affiliation and designation and the contact details of all respondents in the table labelled "Persons met during the situational analysis" at the back of the workbook and not in the concerned section in the workbook.
- Explain the need for confidentiality and how it will be achieved to all the assessment team members, senior MOH officials, and to all respondents.
